# Dual control of liver regeneration by Nr1d1 homeostasis and Klf2 checkpoint

**DOI:** 10.1038/s41420-026-03039-5

**Published:** 2026-04-13

**Authors:** Bingyu Ye, Dejian Xie, Wenlong Shen, Meijuan Yue, Qinpeng Jin, Xinjie Guo, Yan Zhang, Ping Li, Zhihu Zhao

**Affiliations:** 1 Laboratory of Advanced Biotechnology, Beijing Institute of Biotechnology, Beijing, China; 2 State Key Laboratory of Cell Differentiation and Regulation, College of Life Sciences, Henan Normal University, Xinxiang, China

**Keywords:** Nuclear organization, Chromatin

## Abstract

Following extensive liver resections, diminished liver regeneration impairs the maintenance or restoration of sufficient functional liver mass. Currently, effective therapies to restore liver regeneration are lacking, rendering liver transplantation the sole treatment option for end-stage liver disease. Therefore, it is imperative to elucidate the regulatory mechanisms underlying liver regeneration. In this study, we employed a multi-omics approach integrating Hi-C, RNA-seq, and ATAC-seq to dissect the early regulatory mechanisms of liver regeneration in rats and mice. Our results indicate that immune and inflammatory processes are markedly enriched during the early phase of regeneration, accompanied by upregulation of glucocorticoids (GCs) and their receptor (GR). First, the expression dynamics of the GC-related circadian gene Nr1d1 and its regulatory network—including Nfκbiα, Arntl, Clock, and Rora—align with chromatin reorganization, leading us to propose that the GC–GR–Nr1d1 axis is involved in maintaining liver homeostasis. Second, the GR-regulated FoxO family is significantly enriched, and the FoxO-associated gene Klf2 exhibits coordinated changes in expression, chromatin accessibility, and chromatin structure. Functional experiments demonstrate that Klf2 negatively regulates hepatocyte proliferation. Hence, we propose the GC–GR–FoxOs–Klf2 axis acts as a checkpoint in hepatocyte proliferation, preventing premature activation of proliferation- and cell cycle-related genes and ensuring orderly and efficient liver regeneration. Our findings on the role of GCs in liver regeneration may further support their future therapeutic application in liver diseases such as liver fibrosis, alcoholic cirrhosis, and hepatocellular carcinoma (HCC).

## Introduction

Liver regeneration is a well-orchestrated process that allows quiescent hepatocytes to re-enter the cell cycle in response to injury triggers such as toxins, viruses, and acute damage [1]. This capacity is essential for the maintenance or restoration of sufficient functional liver mass. Increasing evidence indicates that liver regeneration is mediated by multiple signaling pathways- including Wnt, Notch, and Hippo- as well as transcription factors such as STAT3, Nrf2, and HNF4α [2]. However, given the multi-step and multi-layered nature of this process, its underlying molecular mechanisms remain incompletely understood.

During the early phase following 2/3 partial hepatectomy (PH), lipopolysaccharide (LPS) stimulates Kupffer cells to release proinflammatory cytokines, including tumor necrosis factor-α (TNF-α) and interleukin 6 (IL-6) [3]. These cytokines play a crucial role in initiating regenerative activity, activating signaling pathways in hepatocytes such as NF-κB, JAK-STAT, PI3K-Akt, and AP1 [4]. Thus, precise control of the inflammatory response is a critical determinant of successful liver regeneration.

The endocannabinoid system (ECS), which is closely involved in inflammation and immune processes, promotes hepatocyte proliferation during liver regeneration by binding to cannabinoid receptors (CBRs) and inducing cell‑cycle proteins such as Forkhead Box M1 (FoxM1) that participate in mitotic progression [5]. Similarly, glucocorticoids (GCs)—potent anti‑inflammatory agents used in treating various immune‑mediated inflammatory diseases (e.g., rheumatoid arthritis, asthma) and liver failure—can also promote the development and progression of metabolic dysfunction‑associated steatotic liver disease (MASLD) [6–9]. Like the ECS, GCs first bind to the cytoplasmic glucocorticoid receptor (GR), which then translocates to the nucleus and acts as a transcription factor. Through transcriptional repression, GR-mediated tethering, and blocking of other transcription factors and signaling molecules—including various NF-κB and AP-1 family members—GR suppresses the inflammatory response [8]. Based on these findings, we propose that, analogous to the ECS, GCs likely play an important role in modulating the early inflammatory response triggered by TNF‑α and IL‑6 during liver regeneration.

REV‑ERBα (encoded by Nr1d1) is a nuclear receptor that functions as part of the circadian clock machinery and regulates metabolic and inflammatory processes [10]. Recent studies have shown that Nr1d1 is involved in the regulation of liver fibrosis, for instance, in a carbon tetrachloride (CCl₄)‑induced mouse model, Nr1d1‑deficient mice exhibited greater susceptibility to fibrosis [11]. Mechanistically, as a transcriptional repressor, Nr1d1 recruits corepressors such as NCoR and HDAC3 to form a complex that binds to RevRE or RevDR2 response elements in the promoters of target genes, thereby regulating their transcription [12]. In the liver, Nr1d1 expression is influenced by both the circadian clock and GC signaling [13]. It has been reported that GR can indirectly interact with the E‑box element in the Nr1d1 promoter via the CLOCK:BMAL1 (ARNTL) complex, leading to suppression of Nr1d1 under stress conditions. In turn, Nr1d1 acts as a critical negative‑feedback regulator of core clock components [14]. Furthermore, Nr1d1 affects the stability and nuclear localization of GR, thereby influencing the expression of GR target genes such as Nfκbiα and Adh1 [10]. Given that Nr1d1 shares functional similarities with the ECS in regulating circadian rhythm, metabolism, inflammation, and energetics [15], we hypothesize that during the early phase of liver regeneration, GCs—upon binding to GR—modulate the inflammatory response and maintain liver homeostasis by regulating Nr1d1 and its related genes (e.g., Nfκbiα, Arntl, Clock, Rora) in response to inflammation.

In mammals, the FoxO transcription factor family comprises FoxO1, FoxO3, FoxO4, and FoxO6, all of which share a highly conserved winged-helix DNA-binding domain (DBD) called the ‘forkhead’ domain [16, 17]. FoxO activity is finely tuned by post-translational modifications (PTMs) such as phosphorylation, acetylation, ubiquitylation and methylation [16, 18]. FoxOs regulate a wide array of biological processes, including immunity, metabolic regulation, cellular and organismal homeostasis, cell cycle control, energy homeostasis, autophagy, redox regulation, aging, cancer, and mitochondrial homeostasis [19–32]. Studies have shown that during liver regeneration, endocannabinoids (ECs) interact with hepatic cannabinoid 1 receptors (CB1R) and promote hepatocyte proliferation by regulating FoxM1 [5, 33]. Similarly, FoxO transcription factors play important roles in liver regeneration. For example, knockout of FoxO3 accelerates hepatocyte proliferation and shortens the regeneration time, indicating that FoxO3 may serve as a target for modulating the pace of liver repair [34]. GCs, upon binding to their receptor GR and subsequent nuclear translocation, have been reported to transcriptionally regulate FoxO1 and FoxO3 [35, 36], Therefore, we propose that—similar to ECs [15]—GCs may participate in the regulation of the FoxO signaling pathway during the early phase of liver regeneration, thereby influencing the progression of liver repair.

Similarly, the Kruppel-like factor (Klf) transcription factor family, which consists of a zinc-finger DNA-binding domain also play pivotal roles in immunity, as well as in cell stemness, differentiation, proliferation, malignancy, metabolism [37–39]. Among them, Klf2, a downstream target of the FoxO transcription factors, has been recognized as a key gene regulator and tumor suppressor [40, 41]. Recent studies indicate that Klf2 helps maintain liver endothelial cell homeostasis and inhibits liver fibrosis and cirrhosis [42, 43]. It has also been reported that Klf2 inhibits hepatocellular carcinoma (HCC) development by interacting with TGF-β signaling [44]. Importantly, as a shear stress-inducible transcription factor, Klf2 modulates flow-sensitive gene expression in endothelial cells, one study showed that endothelial Klf2 negatively regulates liver regeneration upon following injury via induction of activin A [45]. Nevertheless, the expression and functional role of Klf2 in hepatocytes during liver regeneration remain largely unexplored.

In this work, in order to decipher the regulatory mechanisms underpinning liver regeneration, we applied a multi-omics approach. In combination, we performed transcriptome sequencing (RNA-seq), assay for transposase-accessible chromatin with sequencing (ATAC-seq), as well as high-throughput chromatin conformation capture (Hi-C), revealing how chromatin structure and accessibly to it contribute to liver homeostasis and regulation of hepatocyte proliferation during the early stages of liver regeneration. Simultaneously, we also conducted functional analyses to elucidate the roles of key transcription factors in orchestrating these regulatory processes. Similarly to the dual role of the endocannabinoid/CB1R system in liver regeneration and hepatocellular carcinoma (HCC), the ECS represents a potential therapeutic target for both liver repair and disease treatment [15, 33]. Our investigation into the role of GCs in liver regeneration may further support their future application in treating liver diseases, including liver fibrosis, alcoholic cirrhosis, and HCC.

## Results

### Hepatocytes rapidly shift from quiescence to an activated state following PH

**Fig. 1:**
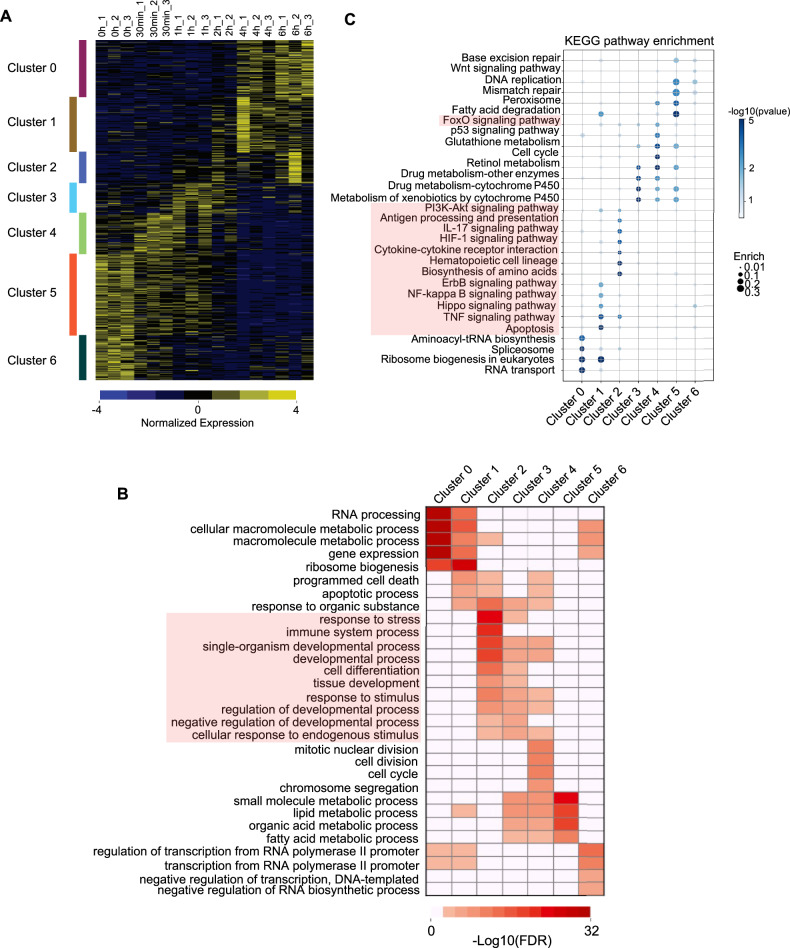
Gene expression patterns, functions, and signaling pathways in the early phase of liver regeneration. **A** Clustering transcriptome results at different time points (0 h, 30 min, 1 h, 2 h, 4 h, and 6 h) in early liver regeneration. For each time point, three independent biological replicates were performed (*N* = 3, three rats in each group). **B** Clustered GO analysis of gene functions corresponding to each of the seven gene clusters. **C** KEGG enrichment analysis of the seven gene clusters. DESeq2 was used to identify DEGs with thresholds of FDR < 0.05 and fold change>2 thresholds, Statistical significance was determined based on an FDR-adjusted *p* < 0.05 (Benjamini–Hochberg).

In the resting adult liver, over 99.9% hepatocytes reside in the G_0_ phase of the cell cycle. However, upon acute liver injury such as PH, the remaining hepatocytes are rapidly activated to proliferate. To elucidate gene expression dynamics during the early phase of liver regeneration, we selected six time points (0 h, 30 min, 1 h, 2 h, 4 h, 6 h) for transcriptomic profiling. First, differentially expressed genes (DEGs) were identified by comparing each time point against the 0 h baseline. Subsequently, k-means clustering categorized these DEGs into seven distinct expression patterns (Fig. 1A). Gene ontology (GO) enrichment analysis revealed that the DEGs are involved in key biological processes, including transcriptional and translational regulation (e.g., negative regulation of transcription, DNA-templated, RNA processing, ribosome biogenesis), metabolic reprogramming (e.g., macromolecule metabolic process), and cell fate control (e.g., apoptotic process, chromosome segregation, cell division) (Fig. 1B). These processes reflect the transition of hepatocytes from quiescence to an activated state shortly after resection. Notably, stress response and immune system processes highlighted in cluster 2 (Red box) represent crucial factors triggered by acute liver injury (Fig. 1B and Supplementary Fig. S1A). KEGG enrichment analysis further highlighted significant enrichment in HIF-1, ErbB, NF-kappa B, IL-17, TNF, and other immune-related signaling pathways (Red box, Fig. 1C and Supplementary Fig. S1B). Together, these results suggest that during early liver regeneration, hepatocytes exit G_0_ and enter the G_1_ phase, accompanied by coordinated expression of functionally diverse genes that collectively support regenerative progression.

We found that in these biological processes, for example, such as drug metabolism, macromolecule metabolic processes, and fatty acid metabolic processes were significantly enriched. These processes have also been documented in the literature to be associated with liver regeneration and are considered key events during liver regeneration [46–48]. Importantly, we also observed that immune- and inflammation- related genes form distinct expression clusters, suggesting their active role in facilitating orderly liver regeneration. We, therefore, focused our subsequent investigation on the functions of genes involved in inflammatory and immune processes.

### Chromatin accessibility dynamics correlate with distinct transcriptional programs

**Fig. 2:**
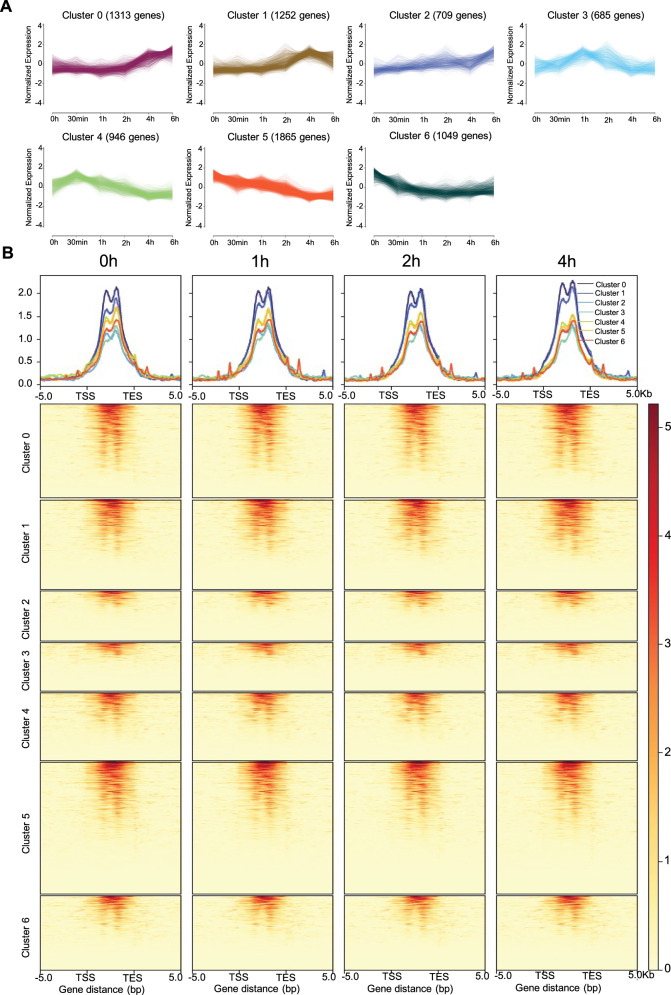
Analysis of chromatin accessibility in different gene expression patterns during the early phase of liver regeneration. **A** The trend of gene expression changes in the seven clusters over regeneration time. DESeq2 was used to identify DEGs with thresholds of FDR < 0.05 and fold change>2 thresholds, Statistical significance was determined based on an FDR-adjusted *p* < 0.05 (Benjamini–Hochberg). **B** The chromatin accessibility profiles of select genes across the seven clusters. The chromatin accessibility changes within the range of 5 kb upstream to 5 kb downstream of genes were analyzed using ATAC-seq data. Peaks were called using MACS2 with default parameters (*q* ≤ 0.05), and DiffBind was run with default parameters, including a minimum overlap of 2 peaks and a false discovery rate (FDR) ≤ 0.05.

Early-phase DEGs during liver regeneration were categorized into seven distinct clusters based on their expression trajectories (Fig. 2A). These clusters included: Cluster 1: characterized by minimal change from 0 to 2 h followed by sustained upregulation from 2 to 6 h. Clusters 3–4: showing rapid upregulation within 0.5–1 h and subsequent downregulation. Clusters 5–6: exhibiting predominant downregulation. We focused on Clusters 1 and 2, which displayed continuous upregulation despite transient fluctuations at 4–6 h. KEGG enrichment analysis confirmed that genes in these clusters were significantly associated with immune and inflammatory regulation (Fig. 1C and Supplementary Fig. S1B).

Given that elevated chromatin accessibility often correlates with enhanced activity, we performed ATAC-seq to examine this relationship in the identified DEG clusters. By assessing chromatin accessibility across gene bodies (defined as regions spanning 5 kb upstream of the TSS to 5 kb downstream of the TES), we observed moderate correlations between gene expression dynamics and locus-specific accessibility changes in all clusters (Fig. 2B and Supplementary Fig. S2). For example, immunity/inflammation-related DEGs in Clusters 1 and 2 exhibited increased expression accompanied by elevated chromatin accessibility (Supplementary Fig. 2S). Together, these results indicate that the dynamics of chromatin accessibility may modulate the transcriptional activity of key signaling pathways during the early phase of liver regeneration.

### Screening key transcription factors in the circadian and FoxO pathway

**Fig. 3:**
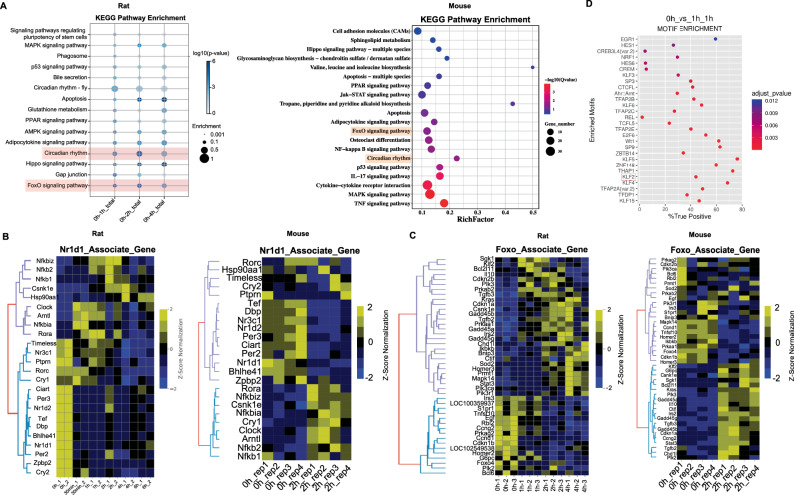
Analysis of circadian rhythm and FoxO signaling pathway-related gene expression and motif enrichment. **A** The overall analysis of KEGG enrichment revealed significant enrichment in circadian rhythm and FoxO signaling pathways (Rat and mouse). **B** Heatmap of gene expression associated with Nr1d1 at different time points during the early regeneration phase (Rat and mouse). **C** Heatmap of gene expression associated with FoxO signaling pathway at different time points during the early regeneration phase (Rat and mouse). **D** Motif analysis revealed a significant enrichment of the transcription factor Klf2’s motif during the early phase of liver regeneration (0h_vs_1h). Enriched motifs with a Benjamini‑Hochberg adjusted *p* < 0.05 were considered significant.

Our findings establish that early-phase gene expression during liver regeneration is closely associated with liver function, regenerative capacity, and locus-specific chromatin accessibility. We further validated these observations by analyzing transcriptomic data from mouse livers at 0 h and 2 h post-PH. Replicating the rat data, analyses revealed a high enrichment of inflammatory and immune-related processes in mice as well (Fig. 3A). To identify key regulators that facilitate adaptation to early regenerative stress, we analyzed pathway activity during this critical period. Analysis revealed significant enrichment of not only typical regeneration pathways (MAPK, PPAR, Hippo) but also circadian rhythm and FoxO signaling in both rodent PH models (Fig. 3A).

Given the established role of Nr1d1 in metabolic, inflammatory, and circadian processes [5], we examined its expression dynamics. Nr1d1 was significantly downregulated during rat and mouse early regeneration (Fig. 3B), while its related genes (Nfκbiα, Arntl, Clock, Rora) displayed inverse expression trends (Fig. 3B). These findings suggest that Nr1d1 may act as a negative regulator, participating in post-PH inflammatory and circadian processes to maintain liver homeostasis.

Concurrently, we also noted marked enrichment of the FoxO signaling pathway and therefore analyzed the expression profiles of FoxO-related genes both in rat and mouse. These genes segregated into two distinct groups: one showing low baseline expression at 0 h followed by continuous upregulation (group 1), and the other exhibiting high initial expression with subsequent downregulation (group 2) (Fig. 3C). Group 1 included genes such as Klf2, Il-10, Tgfb3, Kras (p21), Gadd45a, Gadd45b, and Stat3, whereas group 2 contained genes such as Tnfsf10, Egf, Ccnd1, Foxo4, and Bcl-6 (Fig. 3C). Further analysis indicated that FoxO-related factors regulate multiple target genes involved in cell cycle control, apoptosis, autophagy, oxidative stress resistance and DNA repair, and immuno-regulation, such as Ccnd1, p15, p21, Gadd45, Bcl-6, Bnip3, and Klf2 (Supplementary Fig. S3). Subsequent motif analysis revealed that significant enrichment of the transcription factor Klf2 motif across successive early time points of liver regeneration (Fig. 3D and Supplementary Fig. S4A–B). Based on these observations, we hypothesize Klf2, a factor associated with the FoxO signaling pathway, may play an important role in regulating hepatocyte proliferation during this process.

### GCs affect the expression and chromatin structure of Nr1d1 and its related genes

**Fig. 4:**
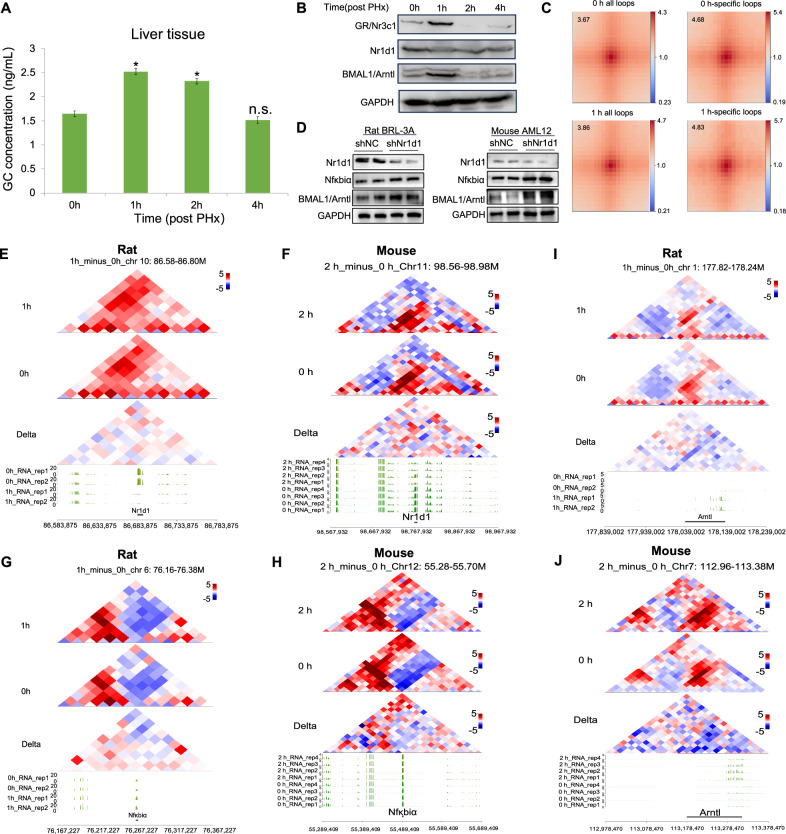
GC-GR-Nr1d1 associated inflammatory and circadian rhythm gene expression and chromatin reorganization. **A** Changes in GC levels during the early stage of liver regeneration. Data are representative of at least three independent experiments. *P < 0.05, **P < 0.01, ***P < 0.001 compared with control; Student’s t-test. **B** Western blot analysis of GR/Nr3c1, Nr1d1, and BMAL1/Arntl expression during the early phase of liver regeneration. **C** Hi-C loop analysis reveals enrichment of circadian rhythm-related genes in “loss” loops (0h_vs_1h). Nr1d1 and circadian genes (e.g., Per3, Nr1d2) were downregulated early in regeneration (Fig. 3B). Motifs for these genes were enriched in “lost” loops. Their enrichment was higher in time point-specific loops (0 h: 4.68; 1 h: 4.83) than in all loops at corresponding times (0 h: 3.67; 1 h: 3.86), and increased from 0 h to 1 h. This demonstrates the specific loss of circadian gene-associated chromatin loops during early regeneration. Chromatin loops were identified using Mustache v0.1.4 at 10-kb resolution with the following parameters: --pThreshold 0.1, --sparsityThreshold 0.88, and --octaves 2. **D** Effects of shNr1d1 on the expression of Nfκbiα and Arntl in rat BRL‑3 A and mouse AML12 cells. **E** The changes in Nr1d1 expression are consistent with its own chromatin interaction frequency (0h_vs_1h) (Rat). **F** The changes in Nr1d1 expression are consistent with its own chromatin interaction frequency (0h_vs_2h) (Mouse). **G** The changes in Nfκbiα expression are consistent with its own chromatin interaction frequency (0h_vs_1h) (Rat). **H** The changes in Nfκbiα expression are consistent with its own chromatin interaction frequency (0h_vs_2h) (Mouse). **I** The changes in Arntl expression are consistent with its own chromatin interaction frequency (0h_vs_1h) (Rat). **J** The changes in Arntl expression are consistent with its own chromatin interaction frequency (0h_vs_2h) (Mouse). Valid chromatin interactions were identified using HiC-Pro v2.11.1 with default parameters.

GCs are steroid hormones known for their anti-inflammatory and immunosuppressive properties. To investigate early changes in the levels of GCs during liver regeneration, we performed ELISA and observed a significant increase in GCs at 1 h and 2 h after PH (Fig. 4A). Correspondingly, we examined the protein expression of the GR/Nr3c1 and found it to be highly elevated at 1 h post-PH (Fig. 4B). Previous studies have reported an inverse correlation between Nr1d1 expression and GR/Nr3c1 activity [14]. Consistent with this, our Hi-C analysis revealed that circadian rhythm-related genes were highly enriched within ATAC “loss” motifs after PH (Fig. 4C). Based on the aforementioned results and analysis, we propose that the binding of GCs to their receptor GR may influence the expression of Nr1d1 and its downstream circadian rhythm-related genes.

Expression analysis showed that rapid downregulation of Nr1d1 following PH coincided with upregulation of Nfκbiα and Arntl (Fig. 3B and Fig. 4B). To further examine the relationship between Nr1d1 and Nfκbiα/Arntl in liver, we silenced Nr1d1 with shRNA in BRL-3A (rat) and AML12 (mouse) hepatocytes. Following Nr1d1 knockdown, protein levels of both Nfκbiα and Arntl was significantly increased (Fig. 4D), suggesting that Nr1d1 is associated with negative regulation of these genes. Next, we assessed whether changes in gene expression were accompanied by alterations in chromatin organization. Hi-C experiments were performed in both rat (at 0, 1, 2, and 4 h post-PH) and mouse (at 0 and 2 h post-PH) liver tissues. Hi-C profiling demonstrated chromatin reorganization in the genomic loci of Nr1d1, Nfκbiα, and Arntl, which aligned closely with their expression dynamics (Fig. 4E–J). In addition, several other circadian-related genes-including Nr1d2, Per3, Rora, Timeless, Nfκbiz, Dbp, and Cry2- also exhibited expression patterns consistent with chromatin structure changes (Supplementary Fig. S5). Collectively, these findings support a close association between Nr1d1 expression, chromatin remodeling, and transcriptional regulation during the early stages of liver regeneration. While our data indicate that GC elevation coincides with changes in Nr1d1 and its related genes, further mechanistic studies will be worthy to determine whether Nr1d1 directly mediates these chromatin alterations.

### Transcriptional changes of transcription factors Klf2 is positively correlated with changes in its chromatin accessibility and chromatin structure

**Fig. 5:**
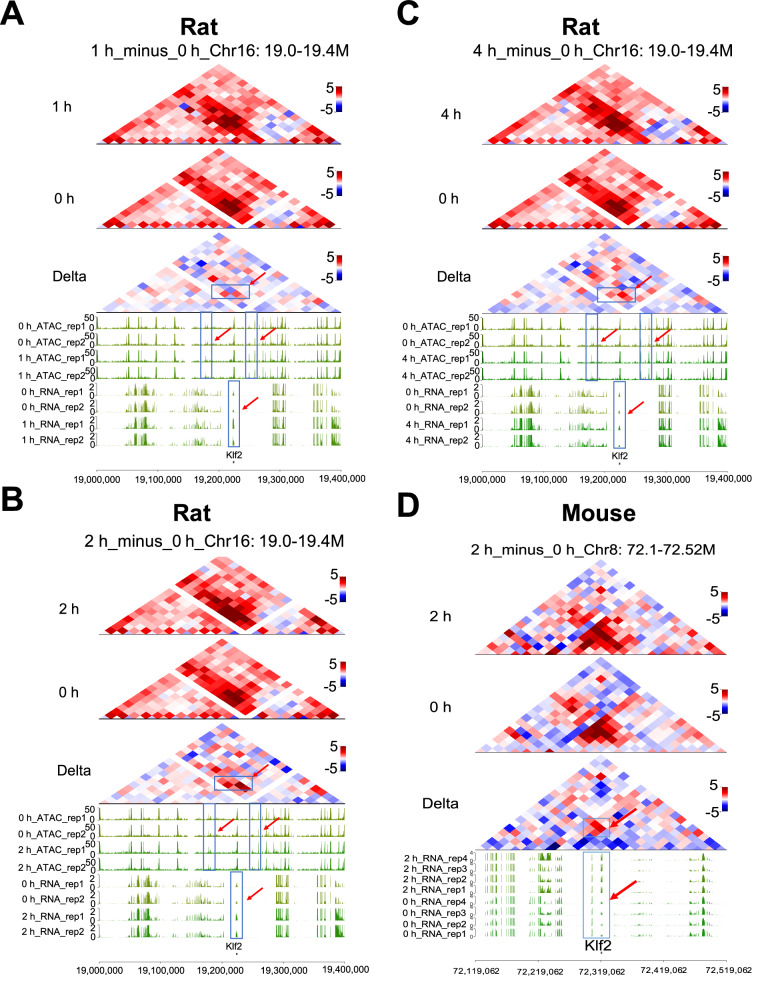
Correlation of Klf2 transcriptional expression, chromatin accessibility, and chromatin structure. **A** Analysis of consistency between the expression of the transcription factor Klf2 and chromatin structure and accessibility (1h_minus_0h) (Rat). **B** Analysis of consistency between the expression of the transcription factor Klf2 and chromatin structure and accessibility (2h_minus_0h) (Rat). **C** Analysis of consistency between the expression of the transcription factor Klf2 and chromatin structure and accessibility (4h_minus_0h) (Rat). **D** Analysis of consistency between the expression of the transcription factor Klf2 and chromatin structure (2h_minus_0h) (Mouse). Red arrows and boxed regions indicate enhancements in Klf2 transcript levels, chromatin accessibility, and chromatin interactions. For each time point, three independent biological replicates were performed of Hi-C (*N* = 3, three rats in each group). Valid chromatin interactions were identified using HiC-Pro v2.11.1 with default parameters.

Gene expression in eukaryotes is regulated at multiple levels, including epigenetic regulation by three-dimensional (3D) chromatin structure, transcription, translation and post-translation [49]. In recent years, the relationship between gene expression and chromatin structure has garnered considerable attention [50]. Here, we further analyzed this relationship for Klf2, a key downstream target gene of the FoxO signaling pathway, using Hi-C data across four time points during early liver regeneration. Transcriptome analysis revealed a dynamic expression pattern of Klf2 (Fig. 3C). Moreover, motif enrichment analysis of DEGs indicated significant enrichment of Klf2-binding motifs near TSSs, suggesting an important role for this transcription factor in early liver regeneration (Fig. 3D and Supplementary Fig. S4A–B). Notably, ATAC-seq profiling demonstrated that chromatin accessibility in regions upstream and downstream of the Klf2 locus changed in concert with its transcriptional activity (Fig. 5A–C), implying that Klf2 expression may be co-regulated by proximal CREs such as promoter and long-range CREs such as enhancer. Further supporting this, Hi-C data revealed that the upregulation of Klf2 in both rodent models correlated with increased chromatin interactions around its gene locus (Fig. 5A–D). Collectively, these findings indicate a strong positive correlation Klf2 transcription and dynamic changes in chromatin accessibility and chromatin structure. In conclusion, by integrating muti-omics data, we have delineated the relationship between transcriptional activity and genome structural reorganization at the Klf2 locus. Our results suggest that chromatin reorganization may be involved in gene transcription as regeneration proceeding. Importantly, together with prior analyses, these data suggest that Klf2, as a key downstream effector of FoxO signaling, may play a functional role in regulating the regenerative process.

### Klf2 knockdown promotes cell cycle progression in normal hepatocytes

**Fig. 6:**
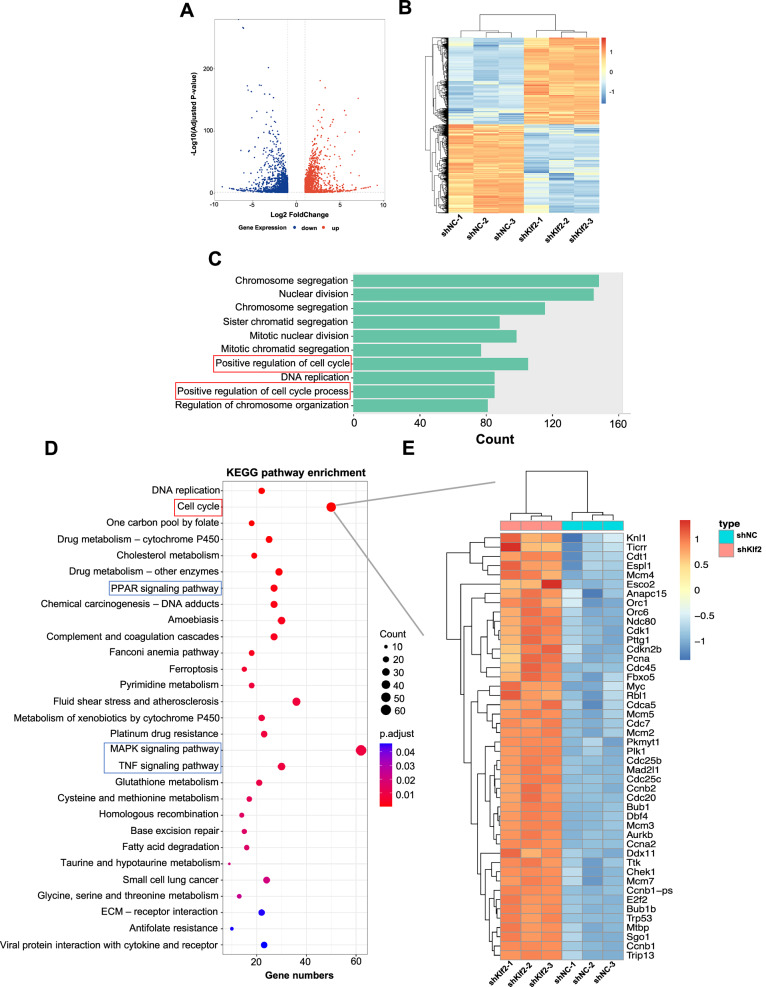
Transcriptome analysis of signaling pathways affected by Klf2 after Klf2 knockdown in AML12 cells. **A** Volcano plot showing fold changes and levels of significance for DEGs (NC_vs_sh). **B** Heatmap representation of the fold changes of DEGs (NC_vs_sh). DESeq2 was used to identify DEGs with thresholds of FDR < 0.05 and fold change>2 thresholds, Statistical significance was determined based on an FDR-adjusted *p* < 0.05 (Benjamini–Hochberg). **C** GO analysis of the biological processes affected by the DEGs (NC_vs_sh). Red box indicates gene sets related to cell cycle and cell cycle process. **D** KEGG pathway enrichment analysis of DEGs (NC_vs_sh) for significantly enriched pathways. The red box indicates significant enrichment of the cell cycle pathway. The blue box indicates significant enrichment of the PPAR signaling pathway, MAPK signaling pathway, and TNF signaling pathway. **E** Heatmap analysis of cell cycle-related genes (NC_vs_sh). For each group, sequencing was performed on three biological replicates.

To further investigate the functional role of Klf2 and its associated signaling pathways, we established a stable Klf2-knockdown AML12 cell line using shRNA, achieving over 60% knockdown efficiency (Supplementary Fig. S6A–B). We then performed transcriptome sequencing on shKlf2 AML12 cells to assess the global impact of Klf2 on cell cycle dynamics and its connection with liver regeneration-related pathways. Bioinformatic analysis identified 2750 DEGs, including 1358 upregulated and 1392 downregulated genes (Fig. 6A, B). GO analysis revealed significant enrichment of biological processes associated with positive regulation of the cell cycle, which was consistent with experimental observations (Fig. 6C). KEGG pathway analysis further indicated strong enrichment of cell cycle-related pathways (Fig. 6D). In addition, several pathways implicated in liver regeneration- such as PPAR, MAPK, and TNF signaling- were also significantly enriched (Fig. 6D). Subsequent focused analysis confirmed pronounced upregulation of cell cycle- related genes (Fig. 6E).

These results demonstrate that Klf2 not only play a critical role in regulating cell cycle progression but also influences multiple signaling pathway associated with regeneration. Our findings suggest that Klf2 may orchestrate regenerative processes through the integrated regulation of interconnected signaling networks and cross-talk mechanisms.

### Klf2 inhibits the expression of cell proliferation and cell cycle-related genes in normal hepatocytes

**Fig. 7:**
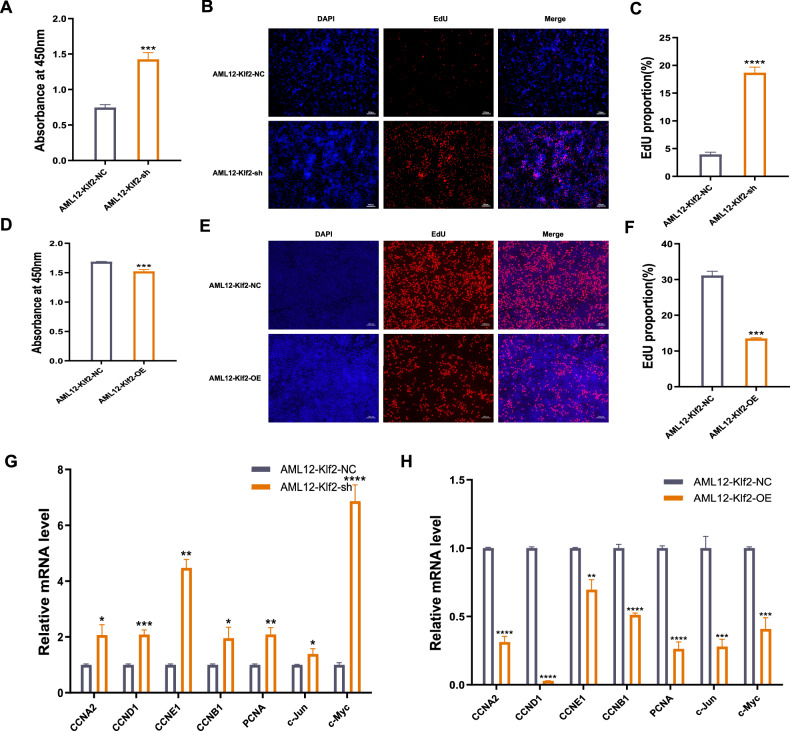
Effects of Klf2 on AML12 cell proliferation and cell cycle progression. **A** CCK-8 assay was performed to determine the viability of shKlf2 in AML12 cells. EdU cell proliferation assay of shKlf2 in AML12 cells (**B**) and the ratio of nuclei with partial EdU incorporation (NC_vs_sh). The scale bar represents 100 μm. **C** Data are representative of at least three independent experiments. **D** CCK-8 assay was performed to determine the viability of Klf2-overexpressing AML12 cells. **E** EdU cell proliferation assay of Klf2-overexpressing AML12 cells. **F** The ratio of nuclei with partial EdU incorporation (NC_vs_OE). The scale bar represents 100 μm. Data are representative of at least three independent experiments. **G** Quantitative real-time PCR detects the relative expression of cell proliferation and cell cycle related genes at the mRNA level after shKlf2. Data are representative of at least three independent experiments. **H** Quantitative real-time PCR detects the relative expression of cell proliferation and cell cycle related genes at the mRNA level after Klf2 OE. Data are representative of at least three independent experiments. *P < 0.05, **P < 0.01, ***P < 0.001 compared with control; Student’s *t*-test.

Transcriptome sequencing analysis indicated that Klf2 knockdown significantly promotes cell cycle progression. To validate this finding, we first examined the effects of Klf2 knockdown and overexpression (OE) on AML12 cell viability. MTT assay showed that shKlf2 markedly enhanced cell viability, whereas Klf2 OE substantially reduced it (Supplementary Fig. S6C–F). Consistent with these results, CCK-8 and EdU assays showed that Klf2 knockdown substantially augmented AML12 cell proliferation, while Klf2 OE suppressed it (Fig. 7A–F). To validate the effect of Klf2 on hepatocyte viability and proliferation, we performed knockdown and OE of Klf2 in rat BRL-3A cells. The results were consistent with those obtained in mouse AML12 cells (Supplementary Fig. S7A–H). These findings collectively demonstrate that Klf2 functions as a negative regulator of hepatocyte proliferation.

We further evaluated the transcriptional impact of Klf2 modulation on proliferation and cell cycle-related gene. Klf2 knockdown significantly elevated the expression of proliferation-related genes such as PCNA, c-Jun, and c-Myc, as well as cell cycle- related genes including CCND1, CCNA2, CCNB1, and CCNE1 (Fig. 7G). Conversely, Klf2 OE suppressed the expression of these genes (Fig. 7F). We obtained similar results in rat BRL-3A cells (Supplementary Fig. S7I–J). Taken together, we reported that Klf2, a key downstream target of the FoxO signaling pathway, serves as a negative regulator of hepatocyte proliferation and cell cycle progression in vitro. This functional role aligns with the “negative regulation of transcription” term identified in our earlier GO analysis (Fig. 1B).

## Discussion

Liver diseases, including non-alcoholic fatty liver disease (NAFLD), liver cirrhosis, and particularly HCC, pose a significant threat to human health. To address these conditions, recent studies using patient‑derived induced pluripotent stem cell (iPSC)‑based hepatocyte models have revealed key bioenergetic defects that drive disease progression in alcohol‑associated cirrhosis [51]. These findings indicate that alterations in cellular energy metabolism contribute to the pathogenesis of cirrhosis, thereby uncovering new potential therapeutic targets. Meanwhile, in cancer immunotherapy, the emergence of immune tolerance presents a major challenge. Encouragingly, strategies such as targeting CD38 have been identified as a promising approach to enhance the efficacy of anti‑PD‑1 therapy, offering new solutions to overcome treatment resistance [52]. Research on liver regeneration provides another vital avenue for treating liver diseases. Although the liver possesses a remarkable capacity to regenerate following PH or chemical-induced injury, this regenerative process is often inadequate in cases of severe acute damage, frequently culminating acute liver failure (ALF) [53]. Therefore, a deeper understanding of the precise regulatory mechanisms of liver regeneration and the identification of strategies to rapidly initiate repair are essential prerequisites for advancing therapies for liver diseases.

This study aims to investigate gene expression and chromatin organization during the early phase of liver regeneration and to clarify the roles of Nr1d1 and Klf2 in glucocorticoid receptor (GR)-mediated regulation of inflammation. Our results indicate that during the early phase of liver regeneration, the upregulation of GCs may affect the expression of Nr1d1, Klf2 and their associated genes, thereby modulating liver homeostasis and proliferation through inflammatory and immune processes. In summary, our findings highlight the critical roles of chromatin reorganization and key regulatory factors in maintaining liver homeostasis and controlling hepatocyte proliferation during early liver regeneration.

In the early phase post-PH, transcriptomic analysis revealed significant enrichment of immune and inflammatory pathways, such as NF-kappa B, IL-17, and TNF signaling, (Fig. 1C). To counteract the impact of excessive inflammation on liver regeneration, the organism activate the relevant anti-inflammatory mechanisms. GCs and GR expression was rapidly upregulated in the early phase (especially within 1 h) of liver regeneration (Fig. 4A–B). Upon ligand binding, GR is transported into the nucleus, where it binds GC-response elements (GREs) near target genes, such as Nfκbiα and alcohol dehydrogenase 1 (Adh1) [10]. Consistent with this, our data show that the upregulation of Nfκbiα expression is accompanied by corresponding changes in its chromatin structure (Fig. 4D, G–H).

Previous studies have also indicated that GR can indirectly suppress Nr1d1 expression during stress by interacting with the CLOCK:BMAL1 complex bound to the E-box in the Nr1d1 promoter [14, 54]. The sharp downregulation of Nr1d1 in the early phase of liver regeneration, coupled with the alleviation of its repressive effects, may represent an additional anti-inflammatory mechanism. Concurrently, the expression of other rhythm genes, including Arntl, Clock, Rora, Cry2, and Per3, was also altered (Fig. 3B). Hi-C analysis further indicated corresponding structural reorganization at the genomic loci of these rhythm-related genes (Fig. 4 and Supplementary Fig. S5). These findings suggest that during the early phase of liver regeneration, GCs may maintain liver homeostasis by affecting the expression of downstream genes involved in inflammation and circadian rhythms. However, the precise mechanism remains to be experimentally validated.

To further investigate the effects of GCs, we observed a significant enrichment of the FoxO signaling pathway—which is influenced by GCs—during the early phase of liver regeneration. The FoxO pathway is known to play important regulatory roles in processes such as cell proliferation and immunity. Previous reports have implicated several downstream targets of the FoxO signaling pathway, such as Gadd45b, Stat3, and Egf, in liver regeneration [55–57]. In seeking new regulatory factors based on FoxO signaling pathway targets, we integrated ATAC-seq and Hi-C data and identified Klf2 as a potential candidate. Although Klf2 in endothelial cells has been reported to negatively regulate liver regeneration via activin A [45], its role in hepatocytes remained unclear. To address this, we overexpressed Klf2 in AML12 and BRL-3A cells and found that it significantly suppressed cell proliferation and cell cycle progression. Transcriptome profiling after Klf2 knockdown in AML12 cells suggested that it influences not only the cell cycle but also several regeneration-associated signaling pathways, including the PPAR, MAPK, and TNF signaling. We propose that Klf2 may coordinately fine-tune the regenerative process by integrating its effects across multiple signaling networks. While our in vitro studies demonstrate Klf2’s role in inhibiting hepatocyte proliferation and implicate potential pathways, the direct downstream targets of Klf2 and the precise molecular mechanisms through which it modulates regeneration warrant further investigation.

In summary, this study leverages multi-omics data (RNA-seq, ATAC-seq and Hi-C) from the early phase of liver regeneration to explore the regulation of liver homeostasis and hepatocyte proliferation. We provide crucial insights into how Nr1d1 may help regulate inflammatory and circadian responses early after injury. Moreover, we identified Klf2 as a negative regulator of hepatocyte proliferation. Future work will focus on delineating the mechanism by which Klf2 restrains premature hepatocyte proliferation, thereby ensuring the orderly progression of liver regeneration. Using rat and mouse liver regeneration models combined with in vitro validation in normal rodent hepatocytes, this study elucidates the role of the GCs-GR-Nr1d1/Klf2 axis in maintaining liver homeostasis and promoting hepatocyte proliferation. Our findings not only deepen the understanding of this regulatory axis in liver regeneration but also highlight its translational potential for developing targeted silencing or agonist strategies against these molecules, with promising clinical applications in human liver diseases such as NAFLD, cirrhosis, and HCC.

## Methods

### Animals

Adult male Sprague Dawley (SD) rats and C57BL/6 J mice (Beijing Vital River Laboratory Animal Technology Co., Ltd., Beijing, China) were housed under specific pathogen-free (SPF) barrier conditions with a 12-h light/dark cycle and provided ad libitum access to food and water. All animal experiments were conducted in accordance with procedures approved by the ethics committee at Henan Normal University, Henan, China, and conformed to the relevant regulatory standards. All animal studies were completed in the experimental animal center of Henan Normal University, China (license number: HNSD-2025-07-07, licensed by the Ministry of Science and Technology of China).

### Two-thirds partial hepatectomy and hepatocyte isolation

At 9–10 weeks of age, rats and mice underwent 2/3 PH using a standardized procedure. Briefly, anesthesia was induced with 3% isoflurane and maintained with 1.5–2% isoflurane. The median lobe and left lateral lobe, which together make up about 2/3 of the liver, were separately tied and resected. Rat hepatocytes were isolated via a 2-step collagenase perfusion method as previously described [58].

### Competitive ELISA for rat GC detection

Anti-rat GC antibody-precoated microplate wells received 50 μL of serum samples or GC standards (varying concentrations). Blank wells were loaded with 50 μL of universal dilution buffer. Subsequently, 50 μL of horseradish peroxidase (HRP)-conjugated antigen working solution was added to each well. The plate was sealed with a cover membrane and incubated at 37 °C for 1 h. After incubation, the liquid was discarded, and each well was washed five times with 300 μL of 1× washing buffer (1 min incubation per wash, followed by thorough removal of liquid and blotting on absorbent paper). Next, 90 μL of 3,3’,5,5’-tetramethylbenzidine (TMB) substrate was added to each well, and the plate was resealed and incubated at 37 °C for 15 min in the dark. The reaction was terminated by adding 50 μL of stop solution per well, and OD was immediately measured at 450 nm. Under HRP catalysis, TMB was converted into a blue intermediate, which then turned yellow upon acidification. The color intensity was inversely proportional to the rat GC concentration in the samples. The GC concentrations were calculated based on the OD values obtained at 450 nm using a microplate reader (BioTek Instruments, Inc., Winooski, VT, USA). All sample were analyzed in triplicate across three independent experiments.

### Cell culture and transfection

Mouse hepatocyte AML12 cells (CRL-2254, ATCC, Manassas, VA, USA) were cultured in AML12 Cell Complete Medium (CM-0602, Procell, Wuhan, China) at 37 °C under 5% CO₂. Rat liver BRL-3A cells (CRL-1442, ATCC, Manassas, VA, USA) were cultured in DMEM (Life technologies, Waltham, MA) containing 10% fetal bovine serum (FBS; Gibco, Grand Island, NY) with 1% penicillin/streptomycin at 37 °C in 5% CO_2_. All cell lines were examined for *Mycoplasma* contamination using the Myco-Lumi Luminescent Detection Kit (C0298S, Beyotime, Shanghai), and none were found to be infected. Klf2 overexpression were generated by cloning full-length cDNAs into pcDNA3.1(+) (empty vector control). Transient transfections employed lipofectamine 2000 (Invitrogen).

For stable knockdown, a Klf2-targeting shRNA sequence: 5′-GACCCTTTCAGTGCC ACTTGT-3′ for mouse or 5′-GCAAGACCTACACCAAGAGTT-3′ for rat was cloned into pLKO.1. Lentivirus was packaged in 293 T cells by co-transfecting pLKO.1-shRNA, psPAX2, and pMD2.G (4:3:1 mass ratio) using polyethylenimine [37]. Viral supernatant was harvested and transduced into AML12 or BRL-3A cells, followed by puromycin selection of stable knockdown populations. Primer sequences for cDNA amplification are provided in Supplementary Table S1 and Supplementary Table S2. As described above, the target shRNA sequences for Nr1d1—specifically 5’- CCAGTACAAACGGTGTCTGAA -3’ for mouse and 5’-GCAAGGGCACCAGCACACATA-3’ for rat—were constructed accordingly.

### MTT assay

Cell viability assessment was performed using MTT assays. AML12 or BRL-3A cells (3 × 10^4^ cells/mL) were seeded in 96-well plates with 100 μL medium and incubated for 24 h at 37 °C/5% CO_2_. At 72 h post-transfection, 20 μL MTT solution (5 mg/mL) was added per well. After 4 h incubation, supernatants were aspirated and 150 μL DMSO added to dissolve formazan crystals. Plates were agitated for 10 min, and absorbance was measured at 490 nm using a BioTek microplate reader (Winooski, VT, USA). Experiments included triplicate wells and three biological replicates.

### CCK-8 assay

DNA synthesis detection employed an EdU kit (RiboBio, Guangzhou). AML12 or BRL-3A cells (3 × 10³ cells/well) were seeded in 96-well plates for 24 h. At 72 h post-transfection, cells were incubated with 100 µL of fresh medium containing 10% CCK-8 reagent for 1 h at 37 °C. Absorbance values of the wells at 450 nm were detected using a microplate reader (BioTek Instruments, Inc., Winooski, VT, USA). Triplicate wells and three independent experiments were performed.

### EdU assay

DNA synthesis detection employed an EdU kit (RiboBio, Guangzhou). AML12 cells (3 × 10³ cells/well) were seeded in 96-well plates for 24 h. At 72 h post-transfection, 50 μM of EdU was added to each well and cultured for additional 2 h. The cells were fixed with 4% formaldehyde for 15 min and treated with 0.3% Triton X-100 for 10 min. After washing with PBS, 100 μl of 1 × Apollo reaction cocktail was added and incubated for 30 min. Subsequently, the nuclei of cells were dyed in DAPI for 10 min and examined under the microscope. Triplicate wells with three replicates were analyzed.

### Total RNA extraction and qRT-PCR

Total RNA was isolated with TRIzol reagent (QIAGEN) and reverse-transcribed into cDNA using GOScriptTM Reverse Transcription System (Promega). The qRT-PCR amplification was performed on a LightCycler® 96 Instrument (Roche) with default program settings. The primer sequences used for qRT-PCR are shown in Supplementary Table S1.

### Total protein extraction and western blot

Total protein extraction and western blot were performed as described previously [59]. The following antibodies were used: anti-Klf2 (1:1500, Immunoway, YN3112), anti-Nr1d1 (1: 10000, Immunoway, YM9061), anti-IκB-α (Nfκbiα) (1:5000, Immunoway, YM8511), anti-BMAL1(Arntl) (1:5000, Immunoway, YM8505), anti-Glucocorticoid Receptor (Nr3c1) (1:2000, Immunoway, YM8180), GAPDH (1:5000, Immunoway, YN5585).

### RNA-seq and data processing

Following quality control, RNA sequencing libraries were prepared using the TruSeq RNA Sample Prep Kit v2 (Illumina, San Diego, CA) and subjected to 150-bp paired-end sequencing on the Illumina Novaseq 6000 platform. Raw data processing was performed as previously described [59]. In brief, paired-end RNA-seq reads were mapped to the rat or mouse reference genome (Rnor_6.0 or mm10) using STAR2.5.3a. DESeq2 v1.24.0 identified differentially expressed genes (DEGs) with thresholds of FDR < 0.05 and fold change>2 thresholds. K-means clustering of gene expression was performed using the k-means function from the sklearn.cluster module in Python. Gene ontology (GO) annotation and Kyoto Encyclopedia of Genes and Genomes (KEGG) pathway analysis of gene were conducted using the clusterProfiler package.

### ATAC-seq and data processing

ATAC-seq was performed using the Omni-ATAC protocol [60]. Briefly, Aliquots of 1 × 10^5^ cells were centrifuged at 500 × *g* for 5 min at 4 °C, washed with chilled PBS, and centrifuged again for 5 min at 4 °C and 500 × *g*. Next, the cell pellet was resuspended in lysis buffer, as set out in the protocol, and cells were left to lyse for 3 min on ice before being centrifuged at 500 × *g* for 12 min at 4 °C. The resulting nuclei pellet was resuspended in transposition mix by pipetting up and down six times. Reactions were incubated at 37 °C for 30 min in a thermomixer with 1000RPM mixing. After transposition, DNA was purified with the MinElute Reaction Clean-up Kit (QIAGEN) and eluted in 10 μL elution buffer. Finally, the library was sequenced on an Illumina Nova6000 platform. Raw reads were processed to obtain clean reads using Fastp (v0.12.4). Cleaned reads were mapped to the rat genome (Rnor_6.0) using Bowtie2 version 2.3.0. All peak calling was performed with MACS2 using “macs2 callpeak —nomodel —keepdup all —call-summits”. Differential binding to peak regions was defined with R package DiffBind with default parameters. Motif enrichment analysis was performed using HOMER (v4.11). Briefly, sequences ±200 bp from the peak summits of each cluster were extracted with the findMotifsGenome.pl script. Known motif enrichment was assessed against GC‑matched background sequences, using the JASPAR core vertebrate database. Enriched motifs with a Benjamini–Hochberg adjusted *p* < 0.05 were considered significant.

### Hi-C and data processing

Hi-C libraries were prepared using the original dilution Hi-C protocol, utilizing the HindIII restriction enzyme [61]. In brief, nuclei were isolated by performing gentle cell lysis on ice for 15 min followed by 30 strokes with a Dounce homogenizer. Nuclei were transferred into ultracentrifugation tubes and resuspended in NEB Buffer 2.1, and digested with 400U of HindIII restriction enzyme overnight at 37 °C with shaking. The next day, the restriction enzyme was heat inactivated and ends were filled in with nucleotides including a biotin-14-dATP and blunt end ligated at 16 °C for 4 h with slow rotation. After Hi-C ligation, the samples were then subject to proteinase K treatment and reverse cross-linking at 65 °C overnight. DNA was purified using phenol-chloroform extraction and ethanol precipitation. Libraries were prepared using Illumina TruSeq adaptors, and biotin containing fragments were isolated using streptavidin coated beads. Libraries were amplified using on-bead PCR amplification and purified using Ampure XP magnetic beads (Beckman Coulter). All of the Hi-C libraries were then sent to a commercial sequencing company (Novogene Co., LTD) and sequenced on the Illumina Novaseq 6000 platform. Following sequencing, raw reads were quality-trimmed using Trimmomatic v0.39. Cleaned reads underwent duplicate removal and were aligned to the rat or mouse reference genome (Rnor_6.0 or mm10) with Bowtie2 v2.3.4.3. Valid chromatin interactions were filtered using HiC-Pro v2.11.1 under default parameters. Topologically associating domains (TADs) were identified at 10-kb and 20-kb resolutions via cWorld’s matrix2 insulation function. Chromatin loops were called at 10-kb resolution using Mustache v0.1.4 with the following parameters: --pThreshold 0.1 --sparsityThreshold 0.88 --octaves 2.

### Statistical analysis

Generally speaking, statistical analysis was performed using GraphPad Prism software (version 8.0), and the data are presented as the mean ± SEM. *P < 0.05 was considered statistically significant and two-tailed Student’s *t* test was used to compare the differences between two groups.

## Supplementary information

Full and uncropped western blots

Supplementary file

Supplementary file 2

## Data Availability

Rat all sequencing raw data (Hi-C, RNA-seq and ATAC-seq) generated in this study have been submitted to NCBI with a BioProject accession number of PRJNA1104701 (http://www.ncbi.nlm.nih.gov/bioproject/1104701). Mouse Hi-C raw data have been submitted to NCBI with a BioProject accession number of PRJNA1417570 (http://www.ncbi.nlm.nih.gov/bioproject/1417570). Mouse RNA-seq raw data have been submitted to NCBI with a BioProject accession number of PRJNA1417571 (http://www.ncbi.nlm.nih.gov/bioproject/1417571).

## References

[1] Matchett KP, Wilson-Kanamori JR, Portman JR, Kapourani CA, Fercoq F, May S, et al. Multimodal decoding of human liver regeneration. Nature. 2024;630:158–65. 38693268 10.1038/s41586-024-07376-2 PMC11153152

[2] Michalopoulos GK, Bhushan B. Liver regeneration: biological and pathological mechanisms and implications. Nat Rev Gastroenterol Hepatol. 2020;18:40–55. 32764740 10.1038/s41575-020-0342-4

[3] Arechederra M, Berasain C, Avila MA, Fernandez-Barrena MG. Chromatin dynamics during liver regeneration. Semin Cell Dev Biol. 2020;97:38–46. 30940574 10.1016/j.semcdb.2019.03.004

[4] Bangru S, Kalsotra A. Cellular and molecular basis of liver regeneration. Semin Cell Dev Biol. 2020;100:74–87. 31980376 10.1016/j.semcdb.2019.12.004 PMC7108750

[5] Mukhopadhyay B, Cinar R, Yin S, Liu J, Tam J, Godlewski G, et al. Hyperactivation of anandamide synthesis and regulation of cell-cycle progression via cannabinoid type 1 (CB1) receptors in the regenerating liver. Proc Natl Acad Sci USA. 2011;108:6323–8. 21383171 10.1073/pnas.1017689108 PMC3076854

[6] Polyzos SA, Targher G. Role of glucocorticoids in metabolic dysfunction-associated steatotic liver disease. Curr Obes Rep. 2024;13:242–55. 38459229 10.1007/s13679-024-00556-1 PMC11150302

[7] Ye C, Li W, Li L, Zhang K. Glucocorticoid treatment strategies in liver failure. Front Immunol. 2022;13:846091. 35371046 10.3389/fimmu.2022.846091 PMC8965693

[8] Auger JP, Zimmermann M, Faas M, Stifel U, Chambers D, Krishnacoumar B, et al. Metabolic rewiring promotes anti-inflammatory effects of glucocorticoids. Nature. 2024;629:184–92. 38600378 10.1038/s41586-024-07282-7

[9] Bazwinsky-Wutschke I, Zipprich A, Dehghani F. Endocannabinoid system in hepatic glucose metabolism, fatty liver disease, and cirrhosis. Int J Mol Sci. 2019;20:2516. 31121839 10.3390/ijms20102516 PMC6566399

[10] Okabe T, Chavan R, Fonseca Costa SS, Brenna A, Ripperger JA, Albrecht U. REV-ERBalpha influences the stability and nuclear localization of the glucocorticoid receptor. J Cell Sci. 2016;129:4143–54. 27686098 10.1242/jcs.190959 PMC5117207

[11] Chen L, Xia S, Wang F, Zhou Y, Wang S, Yang T, et al. m(6)A methylation-induced NR1D1 ablation disrupts the HSC circadian clock and promotes hepatic fibrosis. Pharm Res. 2023;189:106704. 10.1016/j.phrs.2023.106704 36813093

[12] Kim YH, Marhon SA, Zhang Y, Steger DJ, Won K-J, Lazar MA. Rev-erbα dynamically modulates chromatin looping to control circadian gene transcription. Science. 2018;359:1274–7. 29439026 10.1126/science.aao6891 PMC5995144

[13] Inés Pineda Torra VT, Delaunay F, Saladin R, Laudet V, Fruchart J-C, Kosykh V, et al. Circadian and glucocorticoid regulation of rev-erbα expression in liver. Endocrinology. 2000;141:3799–806. 11014236 10.1210/endo.141.10.7708

[14] Murayama Y, Yahagi N, Takeuchi Y, Aita Y, Mehrazad Saber Z, Wada N, et al. Glucocorticoid receptor suppresses gene expression of Rev-erbalpha (Nr1d1) through interaction with the CLOCK complex. FEBS Lett. 2019;593:423–32. 30659595 10.1002/1873-3468.13328

[15] Mukhopadhyay B, Holovac K, Schuebel K, Mukhopadhyay P, Cinar R, Iyer S, et al. The endocannabinoid system promotes hepatocyte progenitor cell proliferation and maturation by modulating cellular energetics. Cell Death Discov. 2023;9:104. 36966147 10.1038/s41420-023-01400-6 PMC10039889

[16] Santos BF, Grenho I, Martel PJ, Ferreira BI, Link W. FOXO family isoforms. Cell Death Dis. 2023;14:702. 37891184 10.1038/s41419-023-06177-1 PMC10611805

[17] Guo X, Peng K, He Y, Xue L. Mechanistic regulation of FOXO transcription factors in the nucleus. Biochim Biophys Acta Rev Cancer. 2024;1879:189083. 38309444 10.1016/j.bbcan.2024.189083

[18] Nakae J, Oki M, Cao Y. The FoxO transcription factors and metabolic regulation. FEBS Lett. 2008;582:54–67. 18022395 10.1016/j.febslet.2007.11.025

[19] Orea-Soufi A, Paik J, Braganca J, Donlon TA, Willcox BJ, Link W. FOXO transcription factors as therapeutic targets in human diseases. Trends Pharm Sci. 2022;43:1070–84. 36280450 10.1016/j.tips.2022.09.010 PMC12194985

[20] Krafczyk N, Klotz LO. FOXO transcription factors in antioxidant defense. IUBMB Life. 2022;74:53–61. 34423888 10.1002/iub.2542

[21] Cheng Z. FoxO transcription factors in mitochondrial homeostasis. Biochem J. 2022;479:525–36. 35195252 10.1042/BCJ20210777 PMC8883485

[22] Sanchez AM, Candau RB, Bernardi H. FoxO transcription factors: their roles in the maintenance of skeletal muscle homeostasis. Cell Mol Life Sci. 2014;71:1657–71. 24232446 10.1007/s00018-013-1513-z PMC11113648

[23] Calissi G, Lam EW, Link W. Therapeutic strategies targeting FOXO transcription factors. Nat Rev Drug Discov. 2021;20:21–38. 33173189 10.1038/s41573-020-0088-2

[24] Jiramongkol Y, Lam EW. FOXO transcription factor family in cancer and metastasis. Cancer Metastasis Rev. 2020;39:681–709. 32372224 10.1007/s10555-020-09883-w PMC7497309

[25] Yadav RK, Chauhan AS, Zhuang L, Gan B. FoxO transcription factors in cancer metabolism. Semin Cancer Biol. 2018;50:65–76. 29309929 10.1016/j.semcancer.2018.01.004 PMC5986595

[26] Murtaza G, Khan AK, Rashid R, Muneer S, Hasan SMF, Chen J. FOXO transcriptional factors and long-term living. Oxid Med Cell Longev. 2017;2017:3494289. 28894507 10.1155/2017/3494289 PMC5574317

[27] Dong XC. FOXO transcription factors in non-alcoholic fatty liver disease. Liver Res. 2017;1:168–73. 30034912 10.1016/j.livres.2017.11.004 PMC6051710

[28] Pan X, Zhang Y, Kim HG, Liangpunsakul S, Dong XC. FOXO transcription factors protect against the diet-induced fatty liver disease. Sci Rep. 2017;7:44597. 28300161 10.1038/srep44597 PMC5353679

[29] Salih DA, Brunet A. FoxO transcription factors in the maintenance of cellular homeostasis during aging. Curr Opin Cell Biol. 2008;20:126–36. 18394876 10.1016/j.ceb.2008.02.005 PMC2387118

[30] Klotz LO, Sanchez-Ramos C, Prieto-Arroyo I, Urbanek P, Steinbrenner H, Monsalve M. Redox regulation of FoxO transcription factors. Redox Biol. 2015;6:51–72. 26184557 10.1016/j.redox.2015.06.019 PMC4511623

[31] Kim S, Koh H. Role of FOXO transcription factors in crosstalk between mitochondria and the nucleus. J Bioenerg Biomembr. 2017;49:335–41. 28417222 10.1007/s10863-017-9705-0

[32] Hornsveld M, Smits LMM, Meerlo M, van Amersfoort M, Groot Koerkamp MJA, van Leenen D, et al. FOXO transcription factors both suppress and support breast cancer progression. Cancer Res. 2018;78:2356–69. 29440168 10.1158/0008-5472.CAN-17-2511

[33] Mukhopadhyay B, Schuebel K, Mukhopadhyay P, Cinar R, Godlewski G, Xiong K, et al. Cannabinoid receptor 1 promotes hepatocellular carcinoma initiation and progression through multiple mechanisms. Hepatology. 2015;61:1615–26. 25580584 10.1002/hep.27686 PMC4406817

[34] Liang CQ, Zhou DC, Peng WT, Chen WY, Wu HY, Zhou YM, et al. FoxO3 restricts liver regeneration by suppressing the proliferation of hepatocytes. NPJ Regen Med. 2022;7:33. 35750775 10.1038/s41536-022-00227-6 PMC9232540

[35] Kuo T, Liu PH, Chen TC, Lee RA, New J, Zhang D, et al. Transcriptional regulation of FoxO3 gene by glucocorticoids in murine myotubes. Am J Physiol Endocrinol Metab. 2016;310:E572–85. 26758684 10.1152/ajpendo.00214.2015 PMC4824139

[36] Qin W, Pan J, Qin Y, Lee DN, Bauman WA, Cardozo C. Identification of functional glucocorticoid response elements in the mouse FoxO1 promoter. Biochem Biophys Res Commun. 2014;450:979–83. 24971545 10.1016/j.bbrc.2014.06.080

[37] Riascos-Bernal DF, Sibinga NE. Neutrophil extracellular traps in cardiac hypertrophy: a KLF2 perspective. J Clin Invest. 2022;132:e147191. 35104806 10.1172/JCI156453 PMC8803320

[38] Shao D, Villet O, Zhang Z, Choi SW, Yan J, Ritterhoff J, et al. Glucose promotes cell growth by suppressing branched-chain amino acid degradation. Nat Commun. 2018;9:2935. 30050148 10.1038/s41467-018-05362-7 PMC6062555

[39] Maity J, Deb M, Greene C, Das H. KLF2 regulates dental pulp-derived stem cell differentiation through the induction of mitophagy and altering mitochondrial metabolism. Redox Biol. 2020;36:101622. 32777717 10.1016/j.redox.2020.101622 PMC7417940

[40] Serr I, Furst RW, Ott VB, Scherm MG, Nikolaev A, Gokmen F, et al. miRNA92a targets KLF2 and the phosphatase PTEN signaling to promote human T follicular helper precursors in T1D islet autoimmunity. Proc Natl Acad Sci USA. 2016;113:E6659–68. 27791035 10.1073/pnas.1606646113 PMC5087025

[41] Salter AI, Ivey RG, Kennedy JJ, Voillet V, Rajan A, Alderman EJ, et al. Phosphoproteomic analysis of chimeric antigen receptor signaling reveals kinetic and quantitative differences that affect cell function. Sci Signal. 2018;11:eaat6753. 30131370 10.1126/scisignal.aat6753 PMC6186424

[42] Sangwung P, Zhou G, Nayak L, Chan ER, Kumar S, Kang DW, et al. KLF2 and KLF4 control endothelial identity and vascular integrity. JCI Insight. 2017;2:e91700. 28239661 10.1172/jci.insight.91700 PMC5313061

[43] Guixe-Muntet S, de Mesquita FC, Vila S, Hernandez-Gea V, Peralta C, Garcia-Pagan JC, et al. Cross-talk between autophagy and KLF2 determines endothelial cell phenotype and microvascular function in acute liver injury. J Hepatol. 2017;66:86–94. 27545498 10.1016/j.jhep.2016.07.051

[44] Li Y, Tu S, Zeng Y, Zhang C, Deng T, Luo W, et al. KLF2 inhibits TGF-beta-mediated cancer cell motility in hepatocellular carcinoma. Acta Biochim Biophys Sin (Shanghai). 2020;52:485–94. 32318691 10.1093/abbs/gmaa024

[45] Manavski Y, Abel T, Hu J, Kleinlutzum D, Buchholz CJ, Belz C, et al. Endothelial transcription factor KLF2 negatively regulates liver regeneration via induction of activin A. Proc Natl Acad Sci USA. 2017;114:3993–8. 28348240 10.1073/pnas.1613392114 PMC5393189

[46] Caldez MJ, Van Hul N, Koh HWL, Teo XQ, Fan JJ, Tan PY, et al. Metabolic remodeling during liver regeneration. Dev Cell. 2018;47:425–38. 30344111 10.1016/j.devcel.2018.09.020

[47] Liu R, Scimeca M, Sun Q, Melino G, Mauriello A, Shao C, et al. Harnessing metabolism of hepatic macrophages to aid liver regeneration. Cell Death Dis. 2023;14:574. 37644019 10.1038/s41419-023-06066-7 PMC10465526

[48] Wang X, Menezes CJ, Jia Y, Xiao Y, Venigalla SSK, Cai F, et al. Metabolic inflexibility promotes mitochondrial health during liver regeneration. Science. 2024;384:eadj4301. 38870309 10.1126/science.adj4301 PMC11232486

[49] Pervouchine D, Popov Y, Berry A, Borsari B, Frankish A, Guigo R. Integrative transcriptomic analysis suggests new autoregulatory splicing events coupled with nonsense-mediated mRNA decay. Nucleic Acids Res. 2019;47:5293–306. 30916337 10.1093/nar/gkz193 PMC6547761

[50] Dai Z, Dai X. Nuclear colocalization of transcription factor target genes strengthens coregulation in yeast. Nucleic Acids Res. 2012;40:27–36. 21880591 10.1093/nar/gkr689 PMC3245921

[51] Mukhopadhyay B, Marietta C, Shen PH, Oiseni A, Mirshahi F, Mazzu M, et al. A patient-based iPSC-derived hepatocyte model of alcohol-associated cirrhosis reveals bioenergetic insights into disease pathogenesis. Nat Commun. 2024;15:2869. 38693144 10.1038/s41467-024-47085-y PMC11063145

[52] Kar A, Ghosh P, Gautam A, Chowdhury S, Basak D, Sarkar I, et al. CD38-RyR2 axis-mediated signaling impedes CD8(+) T cell response to anti-PD1 therapy in cancer. Proc Natl Acad Sci USA. 2024;121:e2315989121. 38451948 10.1073/pnas.2315989121 PMC10945783

[53] Liu R, Cui J, Sun Y, Xu W, Wang Z, Wu M, et al. Autophagy deficiency promotes M1 macrophage polarization to exacerbate acute liver injury via ATG5 repression during aging. Cell Death Discov. 2021;7:397. 34930917 10.1038/s41420-021-00797-2 PMC8688512

[54] Zheng G, Pang S, Wang J, Wang F, Wang Q, Yang L, et al. Glucocorticoid receptor-mediated Nr1d1 chromatin circadian misalignment in stress-induced irritable bowel syndrome. iScience. 2023;26:107137. 37404374 10.1016/j.isci.2023.107137 PMC10316663

[55] Papa S, Zazzeroni F, Fu YX, Bubici C, Alvarez K, Dean K, et al. Gadd45beta promotes hepatocyte survival during liver regeneration in mice by modulating JNK signaling. J Clin Invest. 2008;118:1911–23. 18382767 10.1172/JCI33913 PMC2276398

[56] Hu Z, Han Y, Liu Y, Zhao Z, Ma F, Cui A, et al. CREBZF as a key regulator of STAT3 pathway in the control of liver regeneration in mice. Hepatology. 2020;71:1421–36. 31469186 10.1002/hep.30919

[57] Lopez-Luque J, Caballero-Diaz D, Martinez-Palacian A, Roncero C, Moreno-Caceres J, Garcia-Bravo M, et al. Dissecting the role of epidermal growth factor receptor catalytic activity during liver regeneration and hepatocarcinogenesis. Hepatology. 2016;63:604–19. 26313466 10.1002/hep.28134

[58] Chen T, Oh S, Gregory S, Shen X, Diehl AM. Single-cell omics analysis reveals functional diversification of hepatocytes during liver regeneration. JCI Insight. 2020;5:e141024. 33208554 10.1172/jci.insight.141024 PMC7710279

[59] Ye B, Shen W, Zhang C, Yu M, Ding X, Yin M, et al. The role of ZNF143 overexpression in rat liver cell proliferation. BMC Genom. 2022;23:483. 10.1186/s12864-022-08714-2 PMC9250731 35780101

[60] Grandi FC, Modi H, Kampman L, Corces MR. Chromatin accessibility profiling by ATAC-seq. Nat Protoc. 2022;17:1518–52. 35478247 10.1038/s41596-022-00692-9 PMC9189070

[61] Dixon JR, Selvaraj S, Yue F, Kim A, Li Y, Shen Y, et al. Topological domains in mammalian genomes identified by analysis of chromatin interactions. Nature. 2012;485:376–80. 22495300 10.1038/nature11082 PMC3356448

