# Supplementary material for: Dual control of liver regeneration by Nr1d1 homeostasis and Klf2 checkpoint
Source: Cell Death Discov. 2026 Apr 13;12:224. doi: 10.1038/s41420-026-03039-5 (PMC13183961; doi:10.1038/s41420-026-03039-5)

Figure 4B

GR/Nr3c1


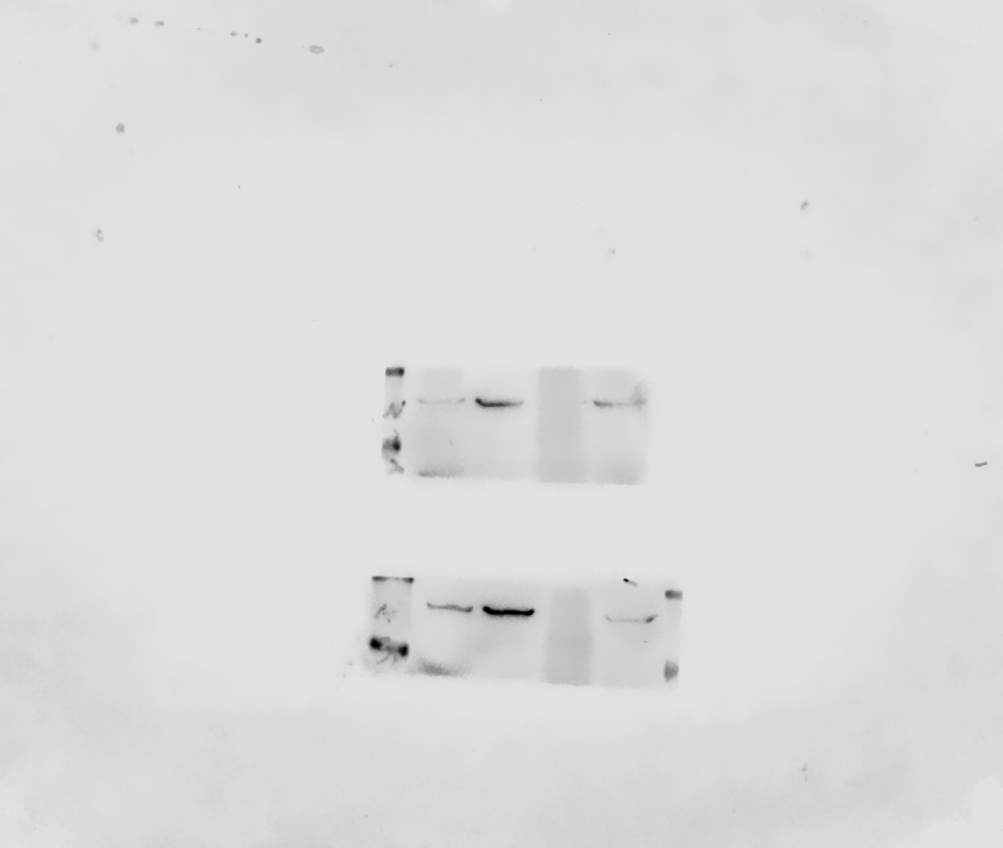


Nr1d1


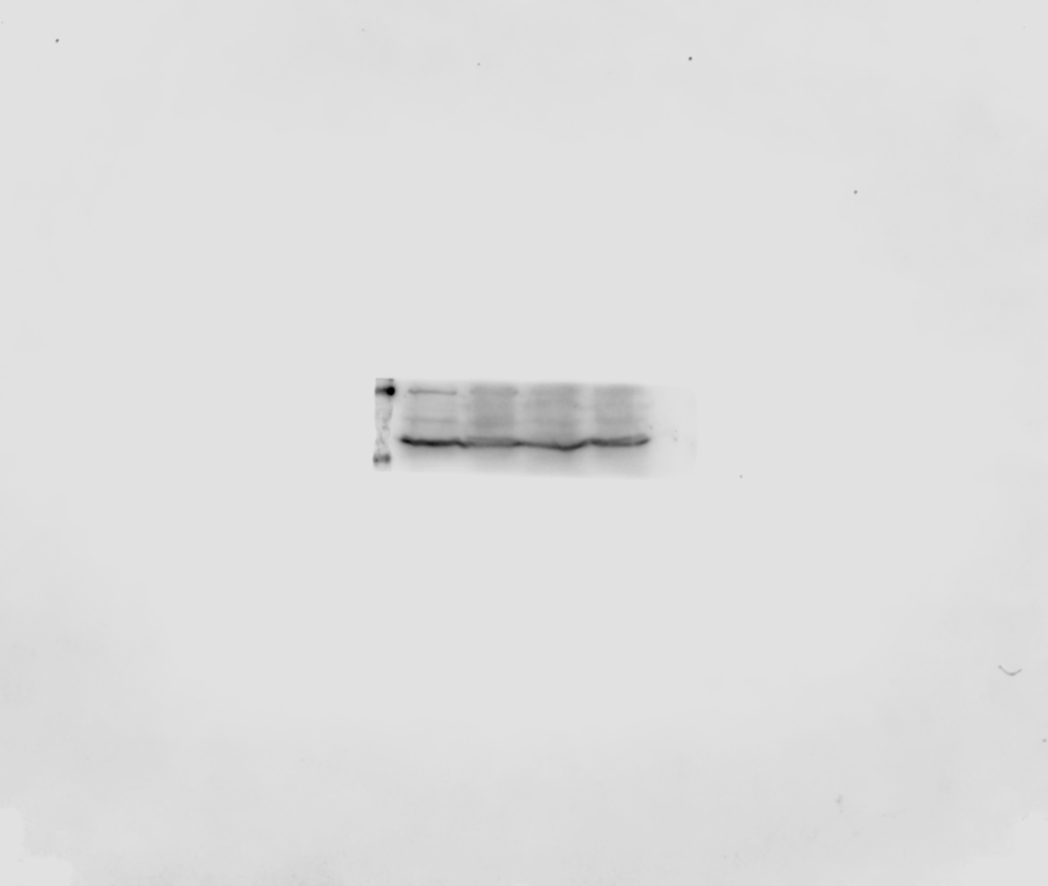


BMAL1/Arntl


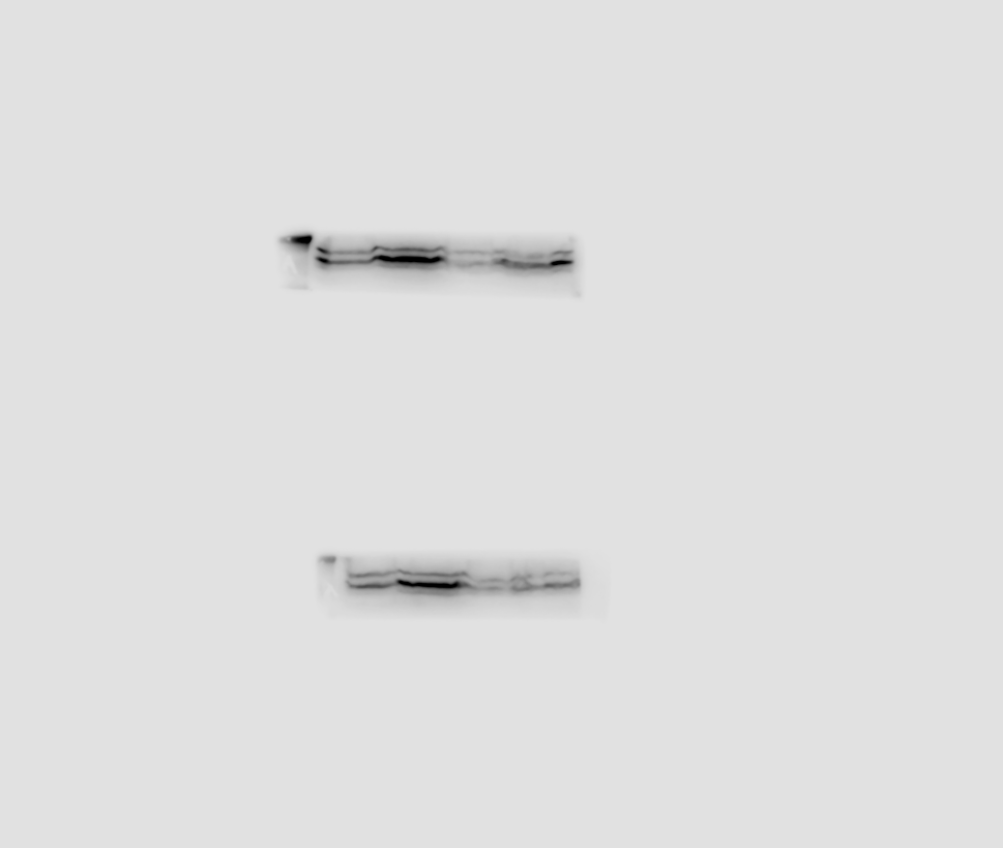


GAPDH


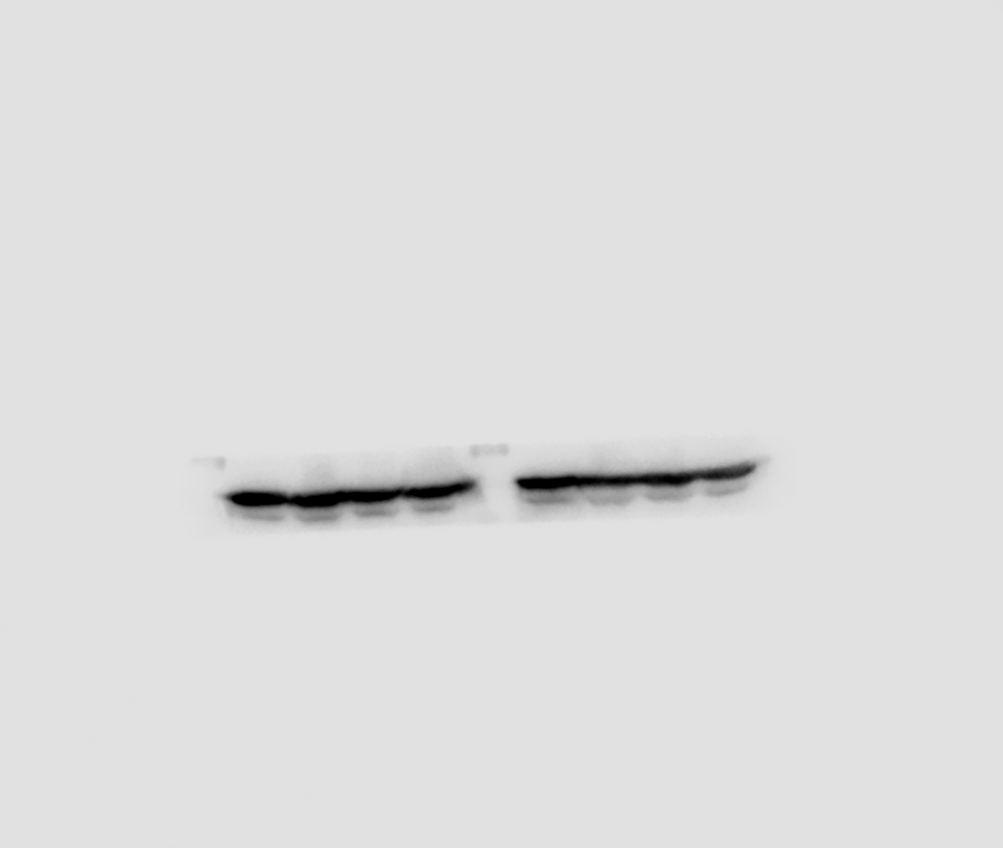


Figure 4D

BRL-3A-Nr1d1


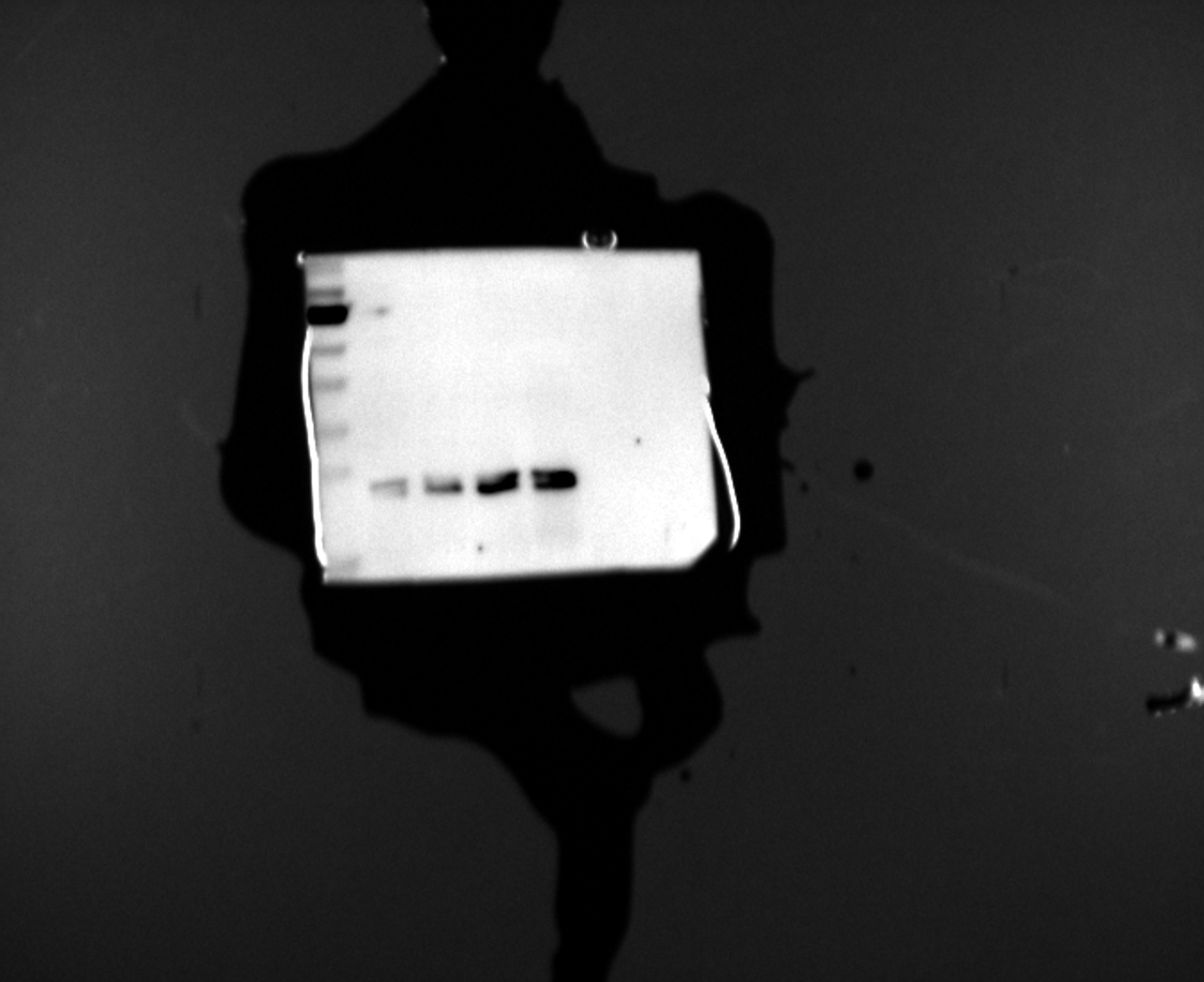


BRL-3A-Nfkbia


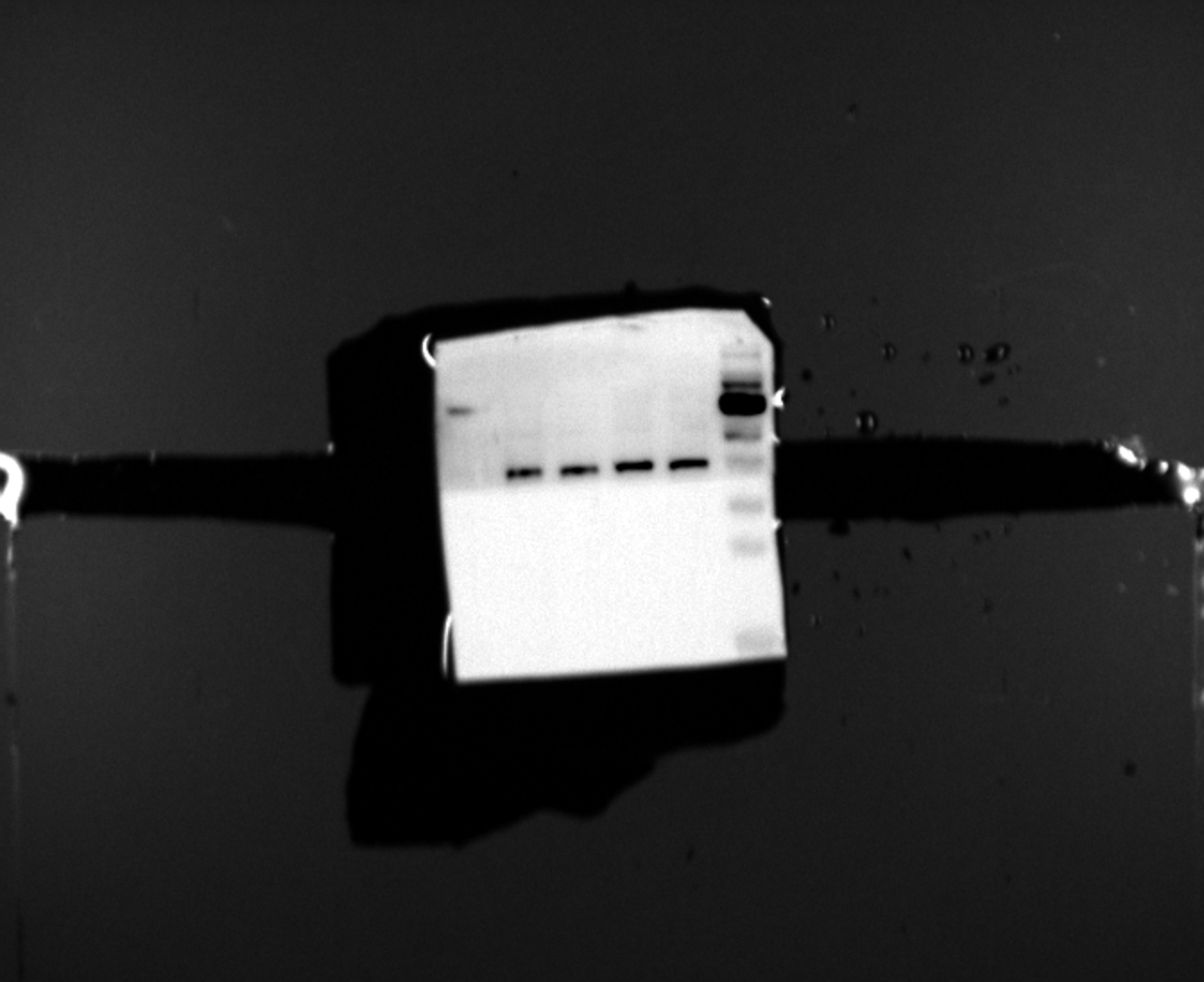


BRL-3A-BMAL1/Arntl


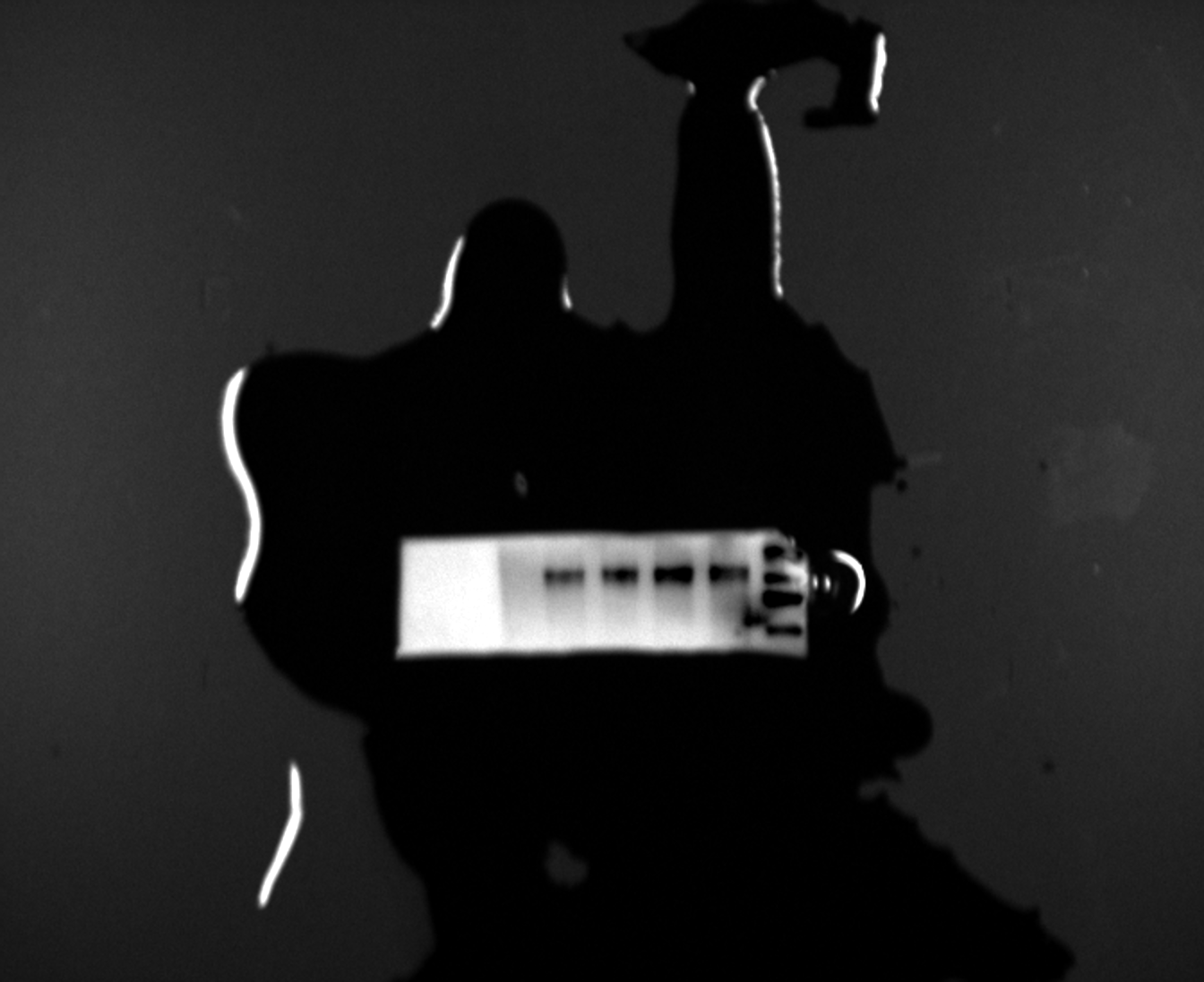


BRL-3A-GAPDH


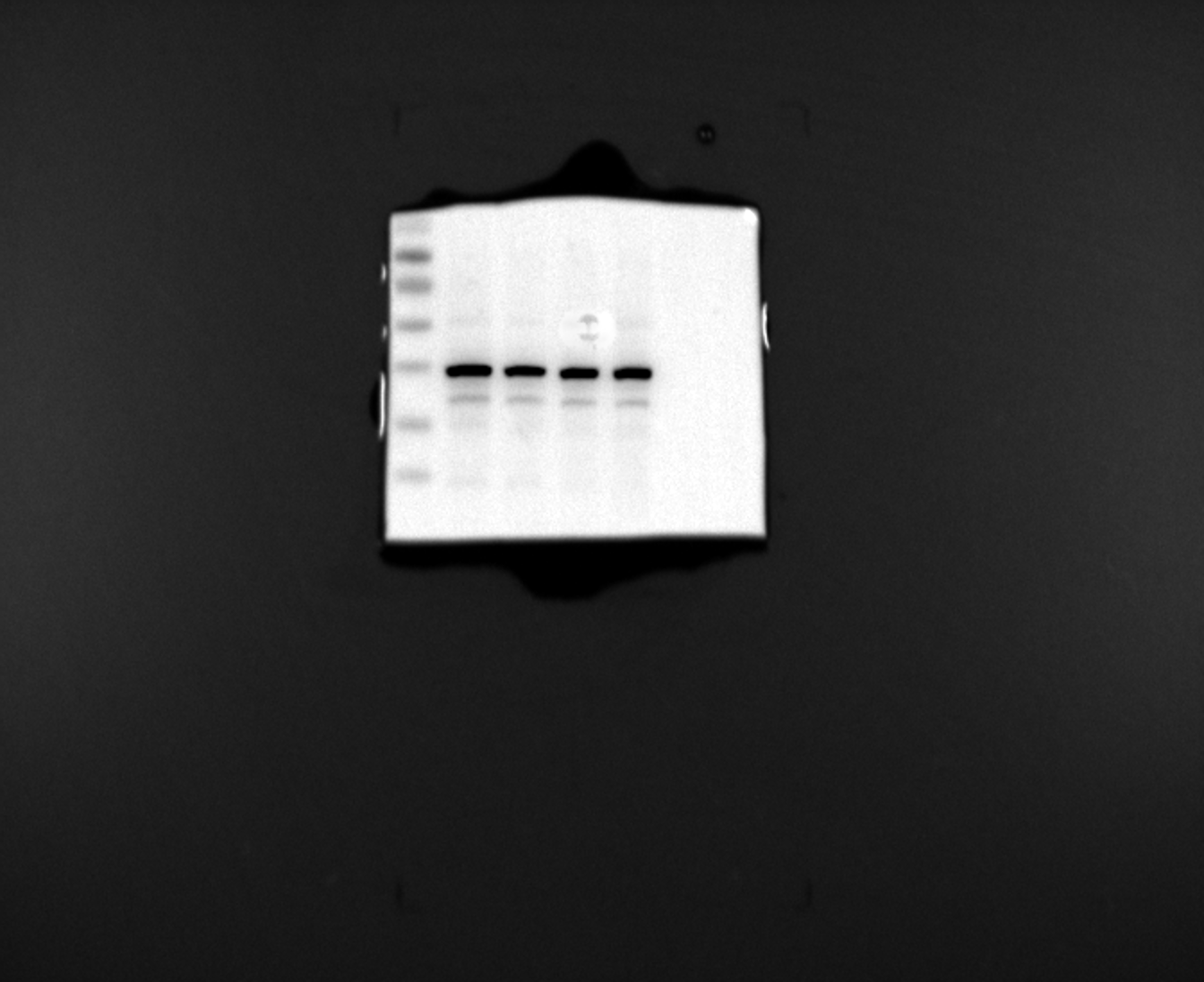


AML12-Nr1d1


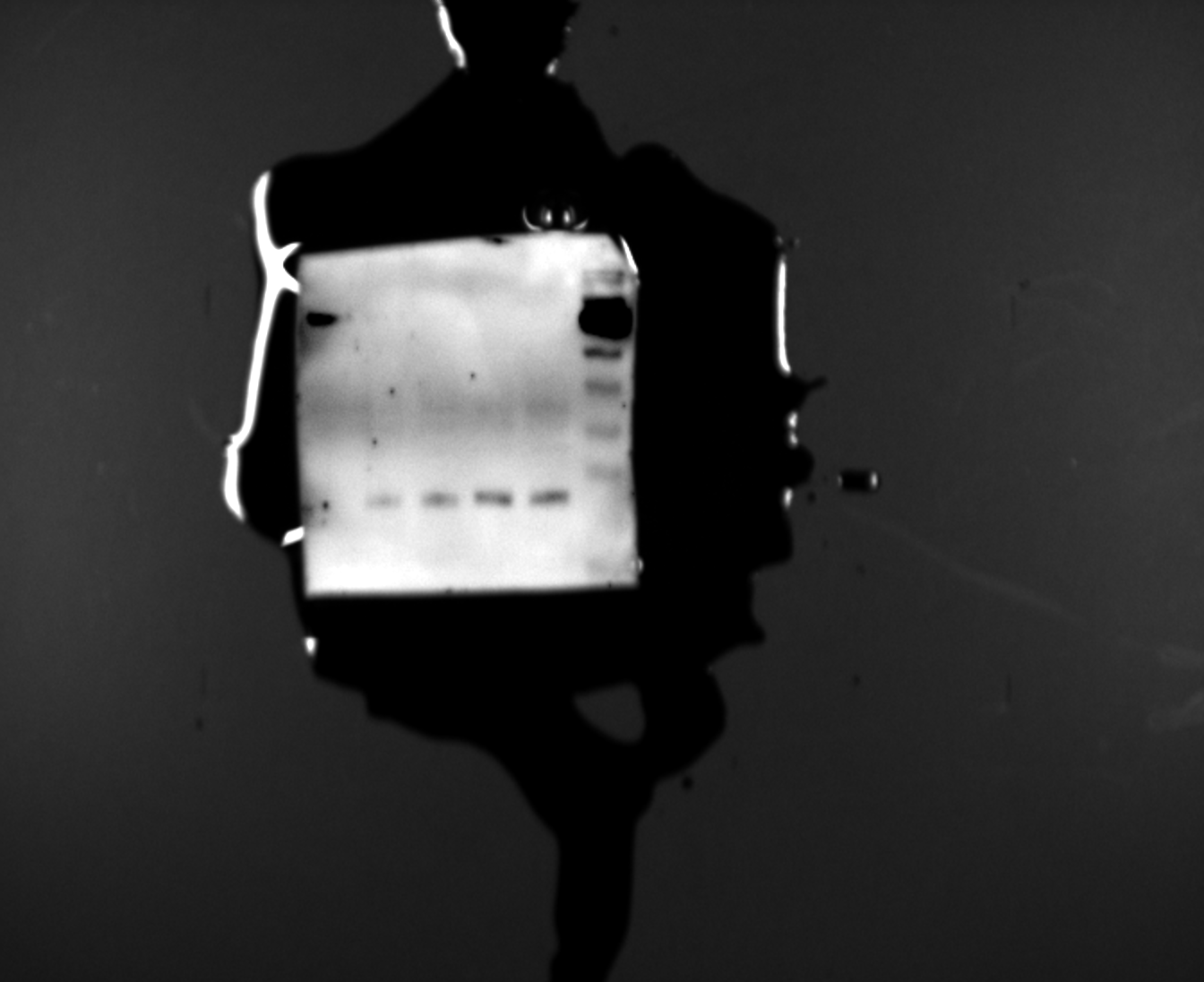


AML12-Nfkbia


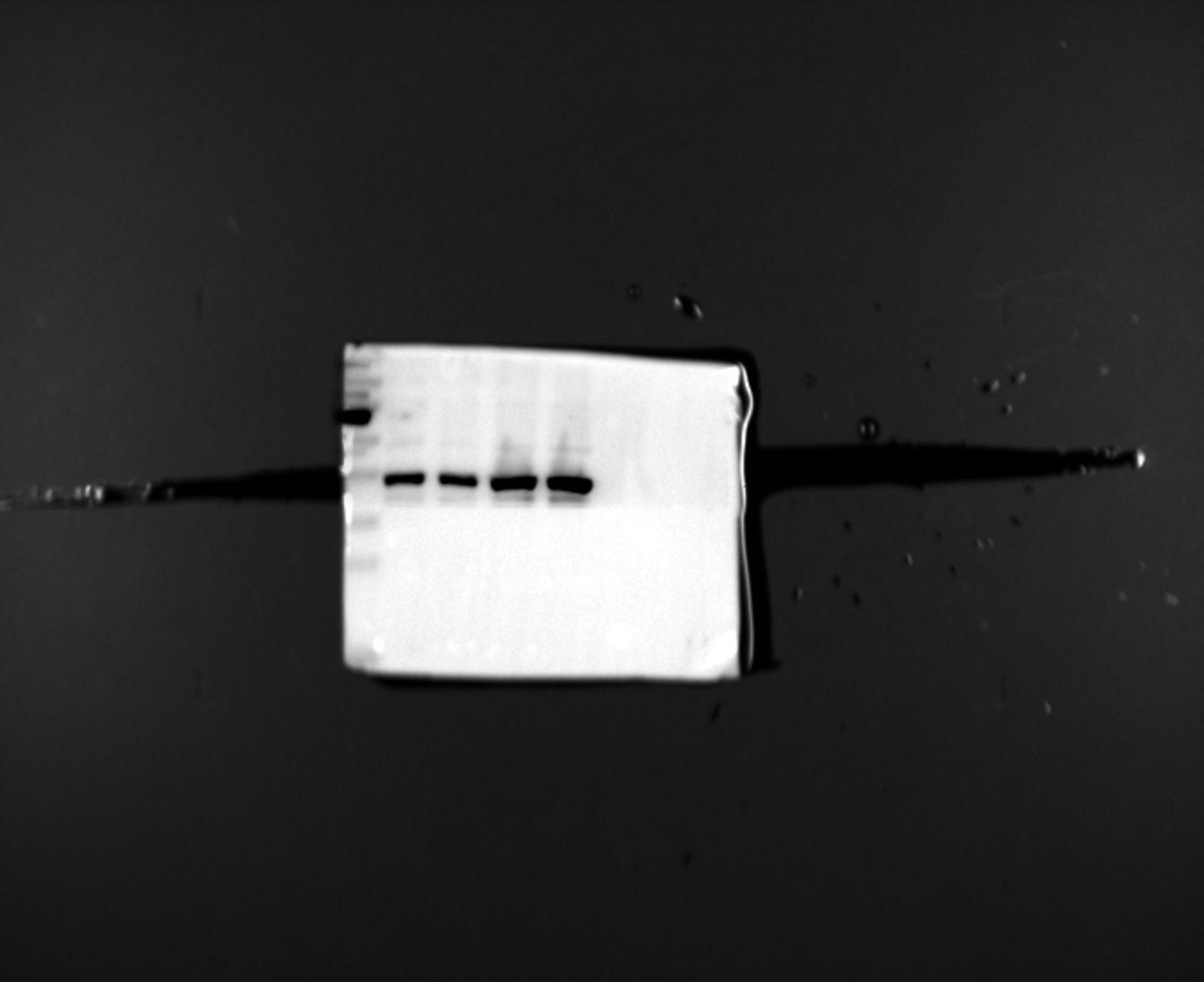


AML12-BMAL1/Arntl


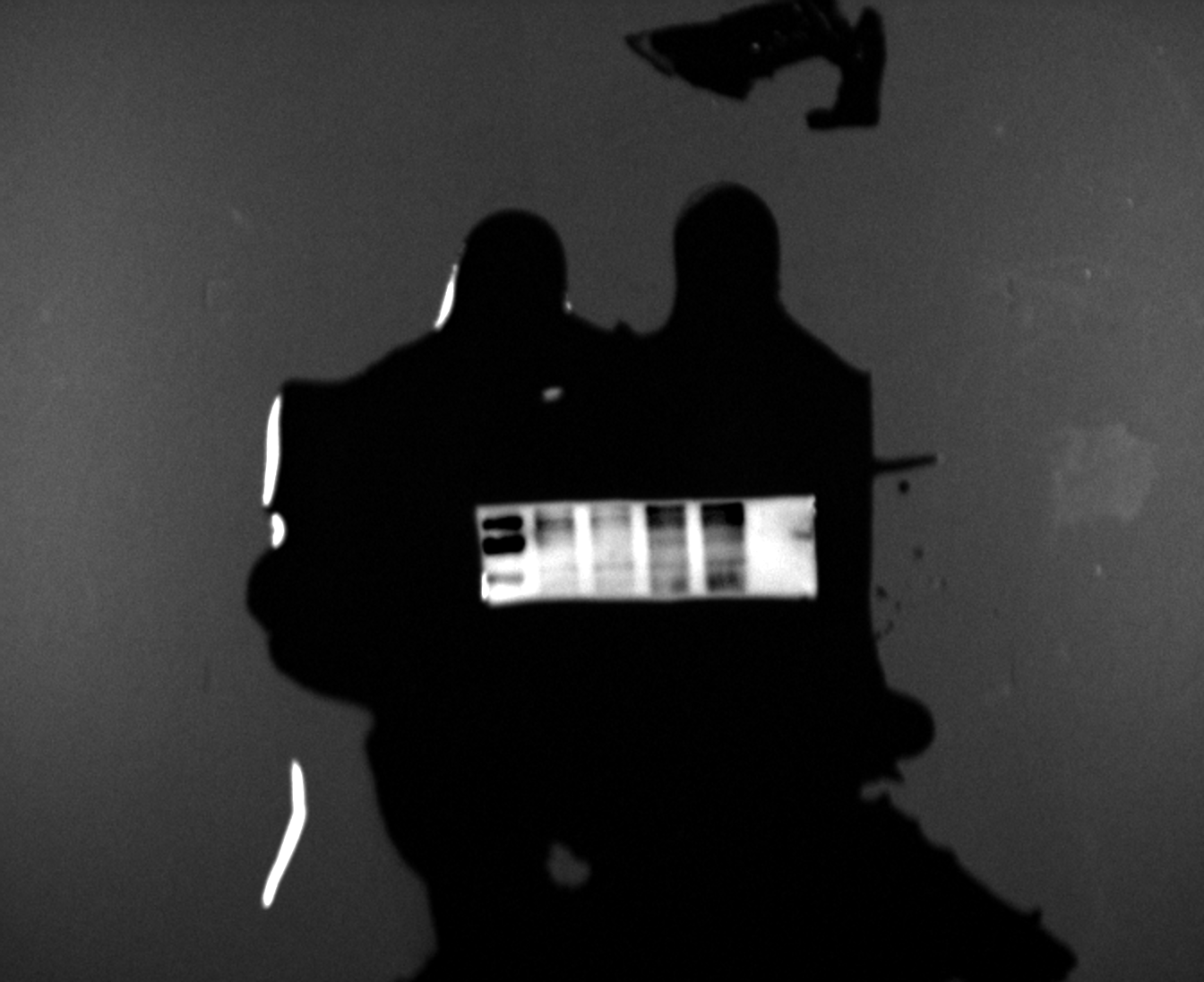


AML12-GAPDH


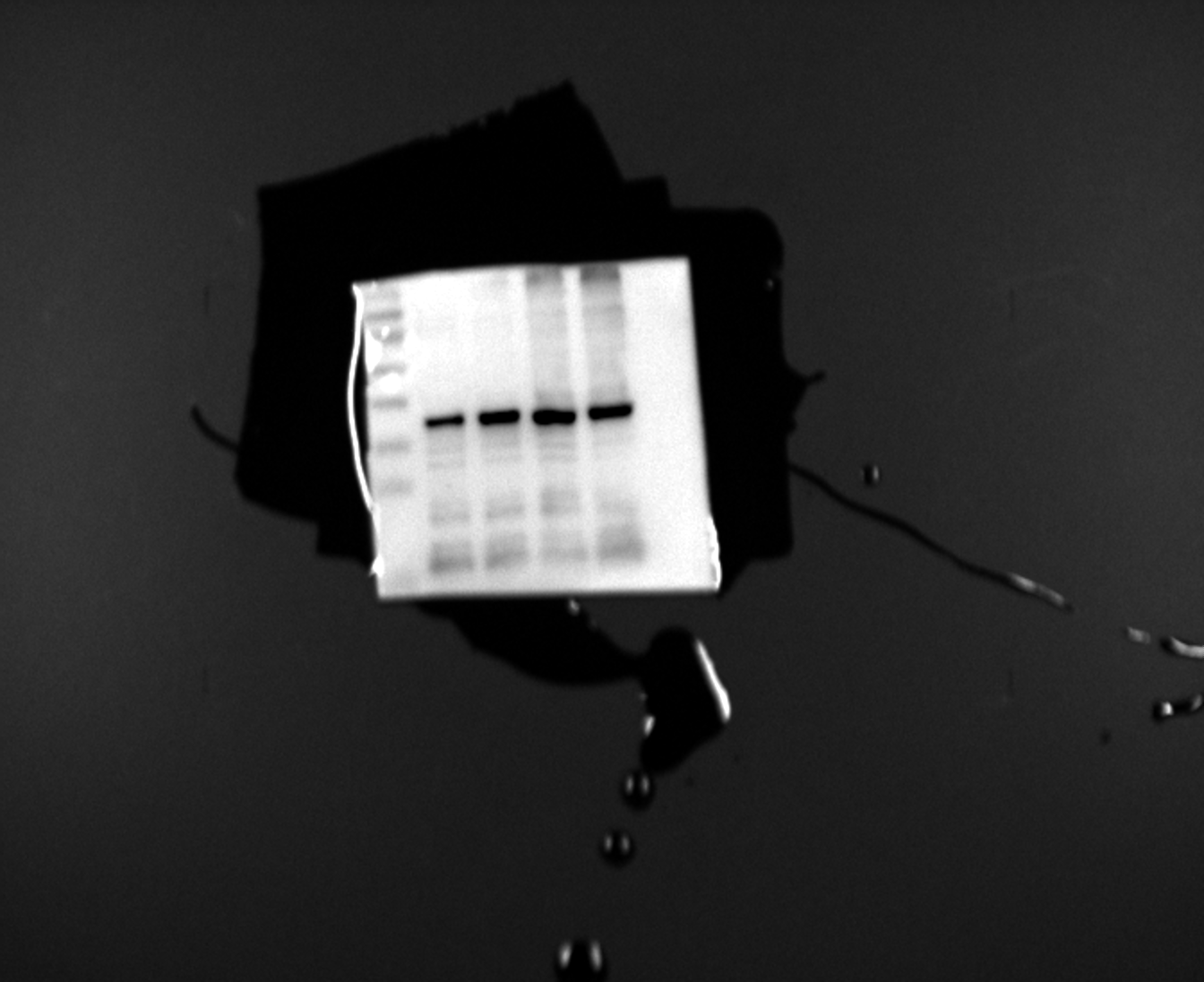


Figure S6B

Klf2


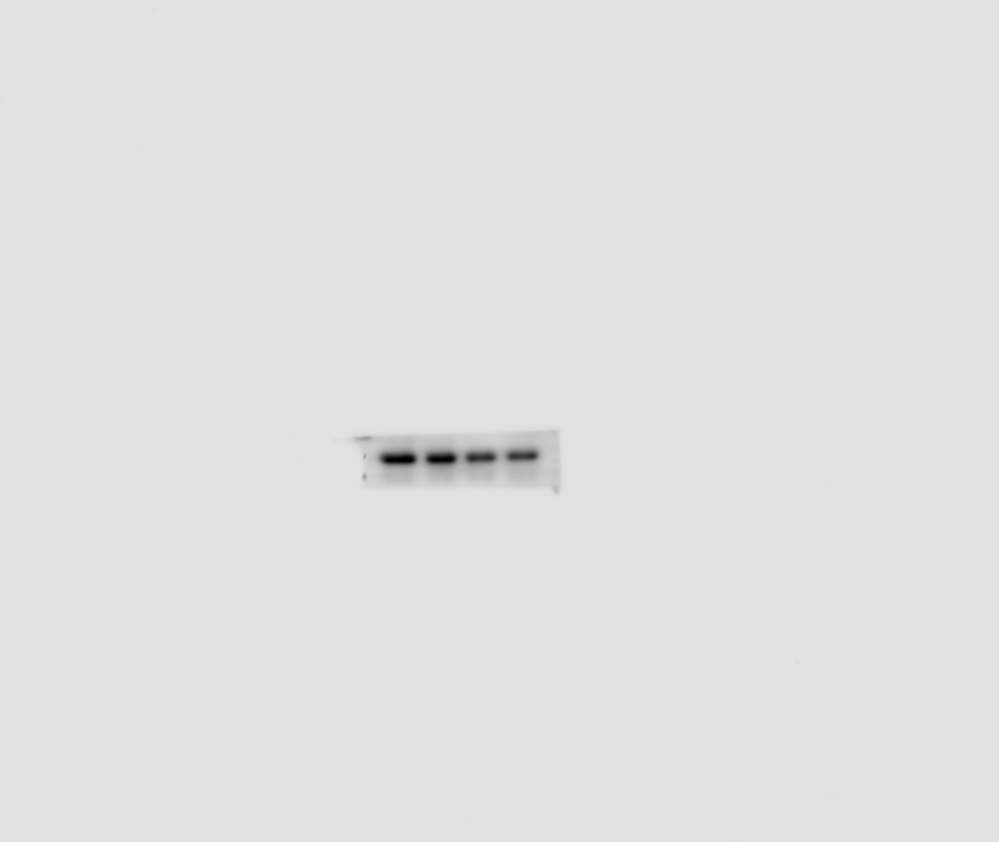


GAPDH


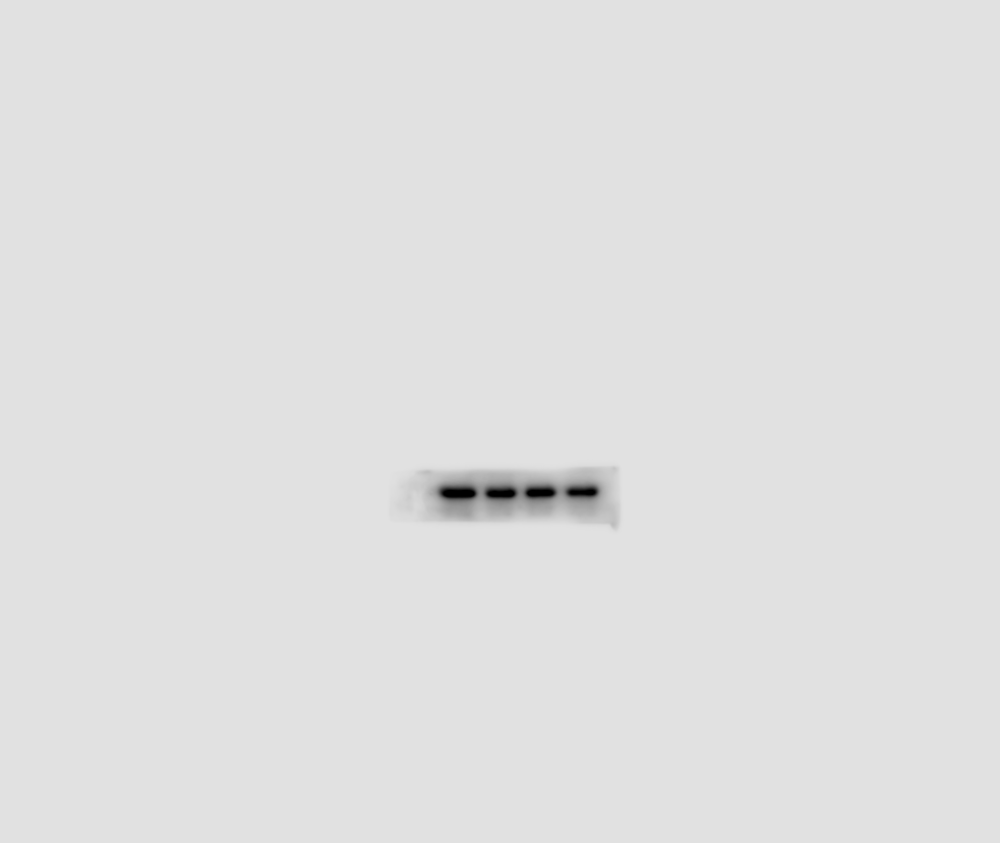


Figure S6E

Klf2


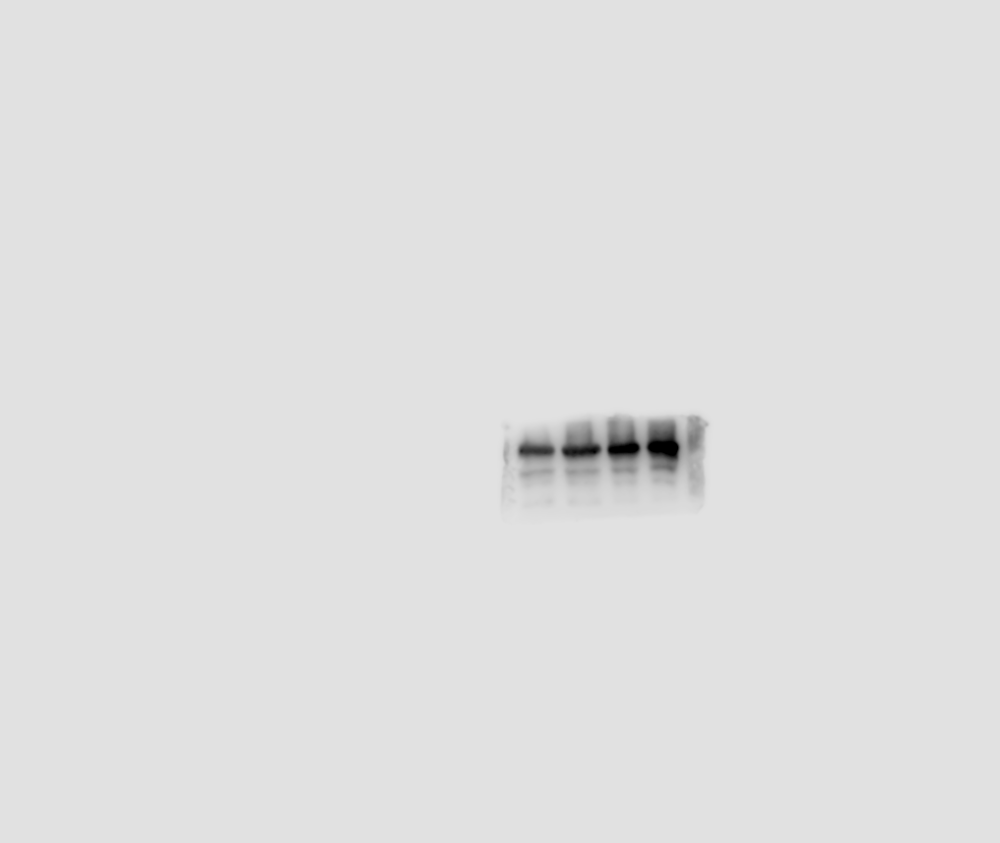


GAPDH


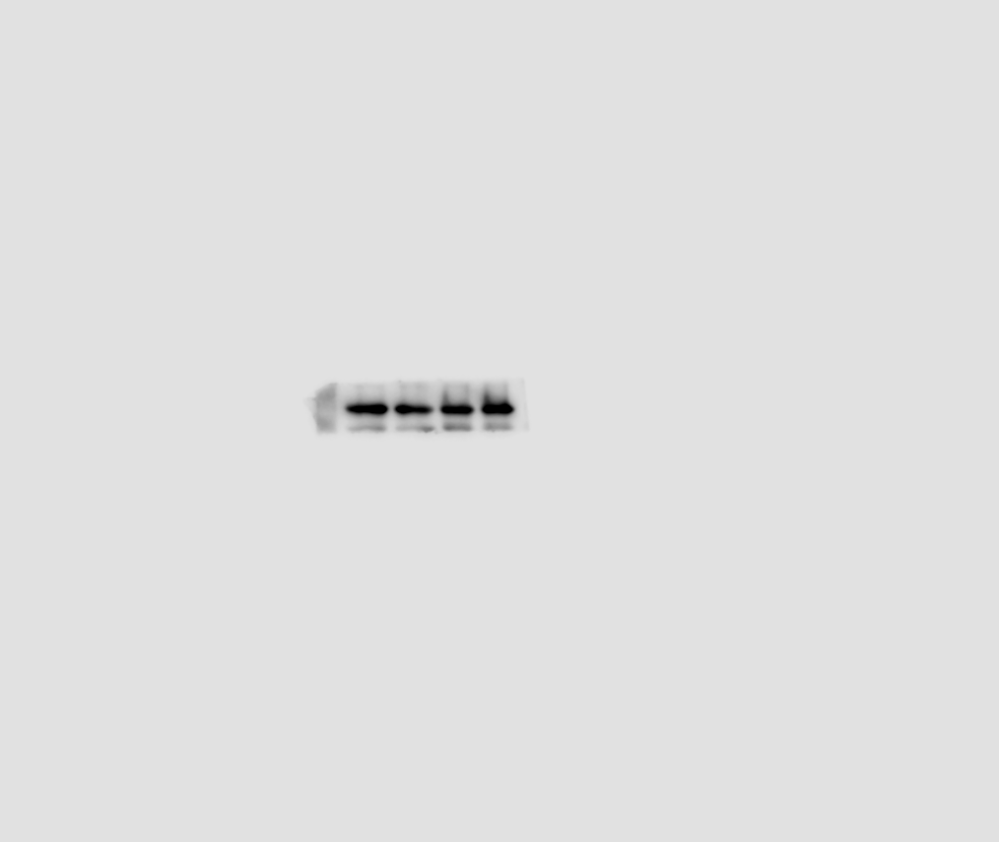


Figure S7B

Klf2


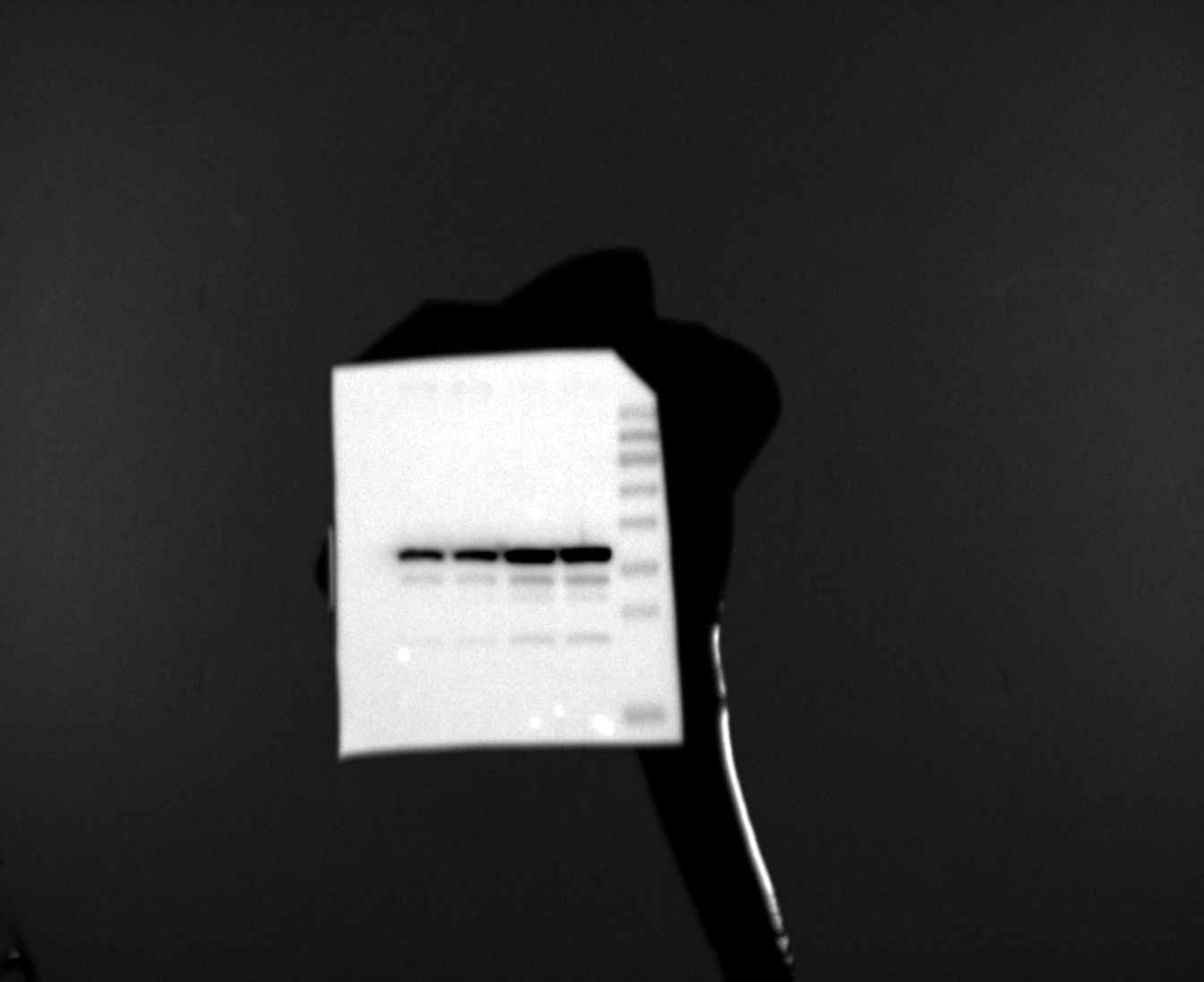


GAPDH


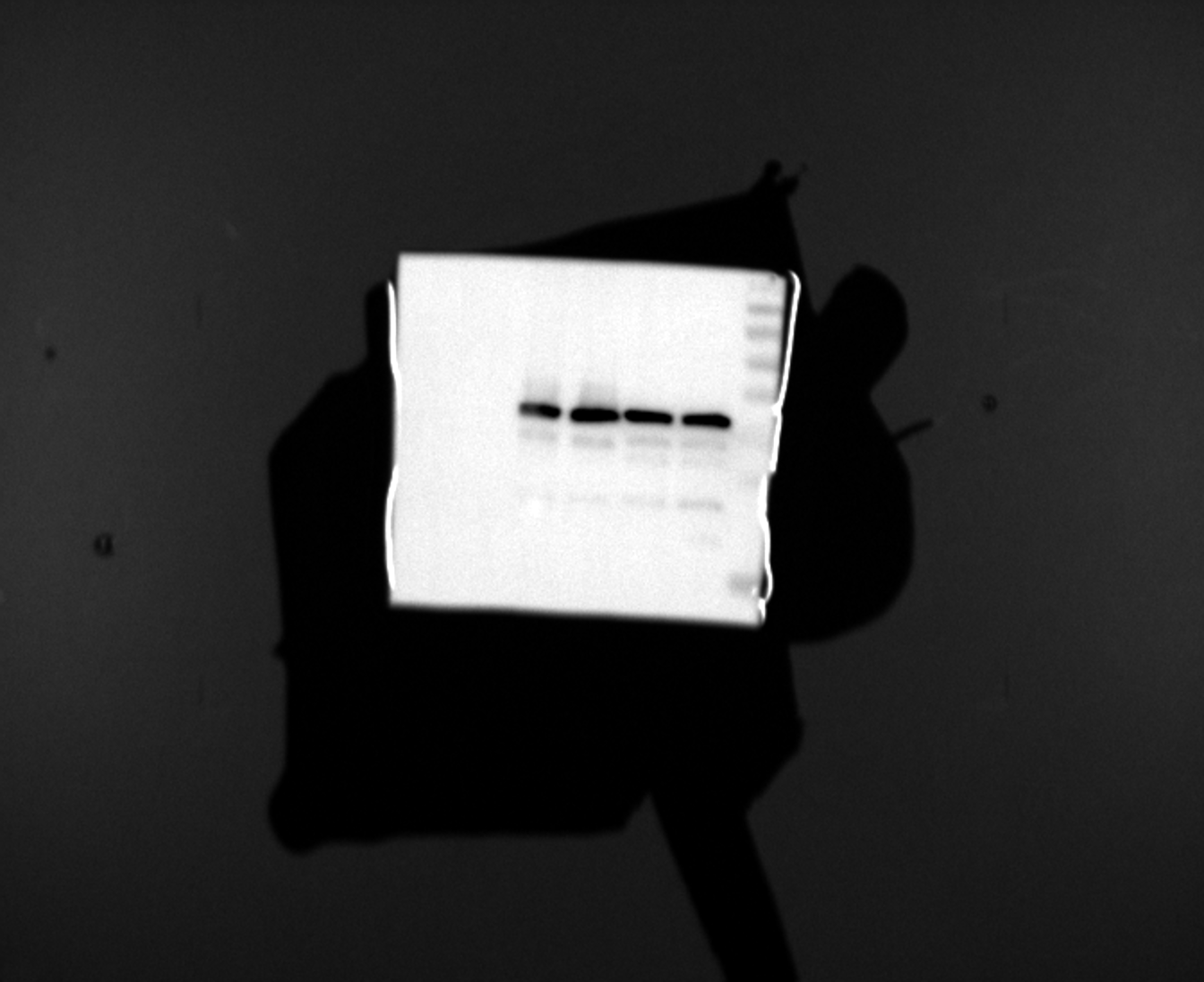


Figure S7F

Klf2


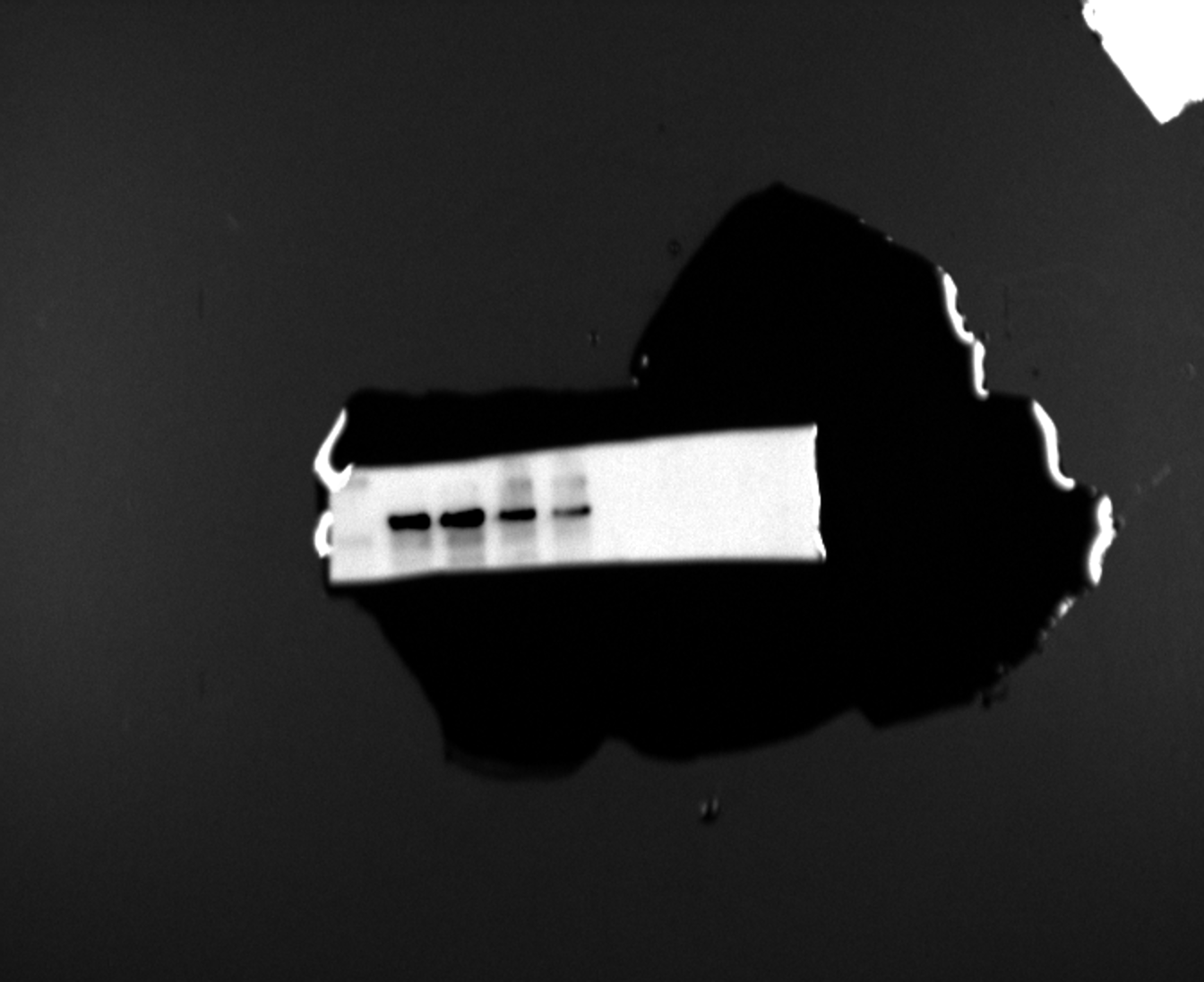


GAPDH


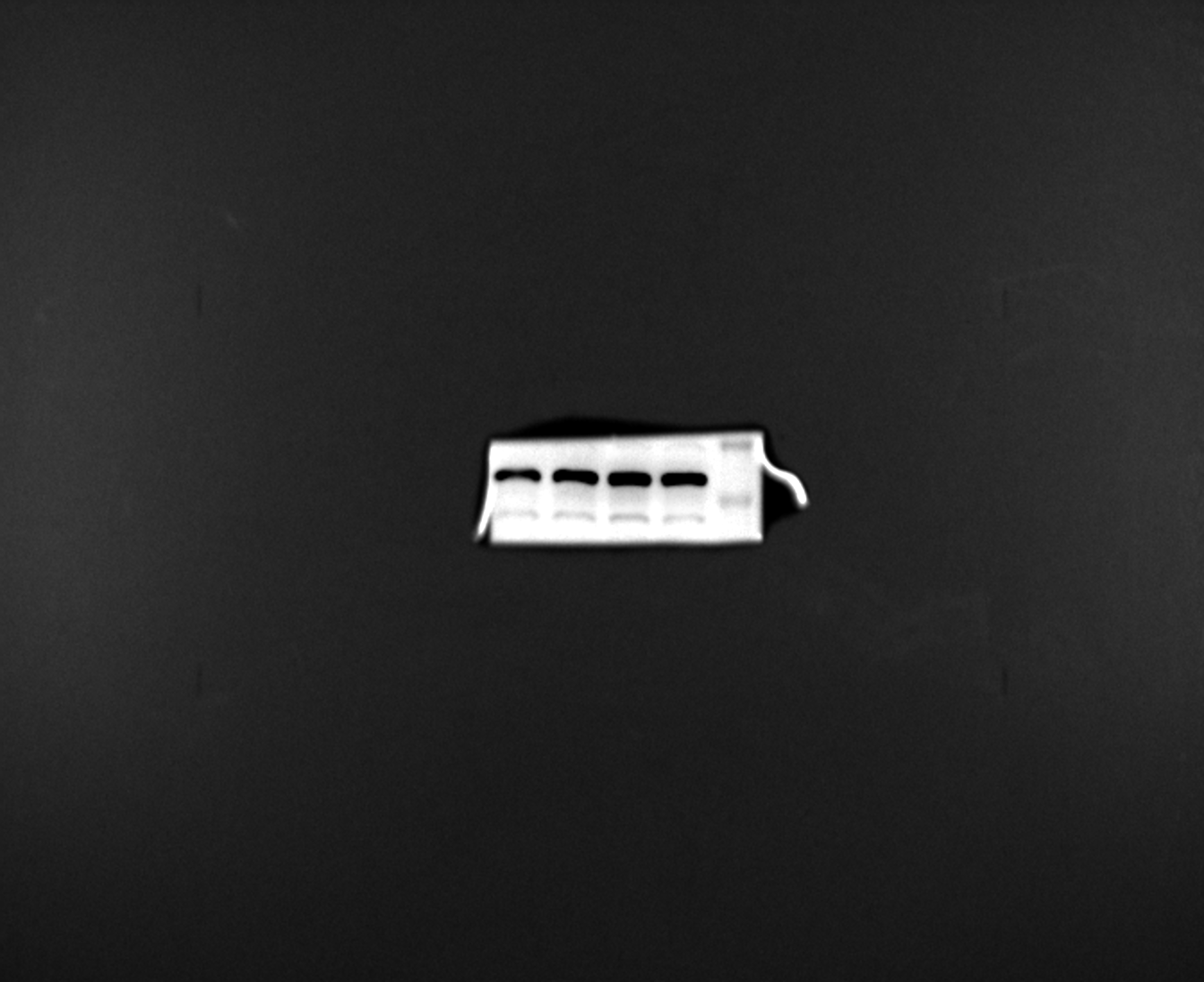

Supplement: Supplementary file 1 — Full and uncropped western blots [file 41420_2026_3039_MOESM1_ESM.docx]
