# Supplementary material for: Dual control of liver regeneration by Nr1d1 homeostasis and Klf2 checkpoint
Source: Cell Death Discov. 2026 Apr 13;12:224. doi: 10.1038/s41420-026-03039-5 (PMC13183961; doi:10.1038/s41420-026-03039-5)
Supplement: Supplementary file 2 — Supplementary file [file 41420_2026_3039_MOESM2_ESM.docx]

**Supplementary Information**

**Table S1** Primers used Klf2 (mouse) full-length cDNA amplification, and qRT-PCR

| Name | Sequence（5' to 3'） |
| --- | --- |
| Klf2-F (OE mouse) | CCCAAGCTTGCCACCATGGCGCTCAGCGAGCCTATCTTG |
| Klf2-R (OE mouse) | CGCGGATCCCTACATGTGTCGCTTCATGTG |
| Klf2-F (m) | GCGTACACACACAGGTGAGA |
| Klf2-R (m) | GTGTCGCTTCATGTGCAAGG |
| c-Myc-F (m) | ATGTTGCGGTCGCTACGTC |
| c-Myc-R (m) | AGAAGTTGCCACCGCCG |
| c-Jun-F (m) | CGATGCCCTCAACGCC |
| c-Jun-R (m) | CTTAGGGTTACTGTAGCCGTAGGC |
| PCNA-F (m) | GAACCTCACCAGCATGTCCA |
| PCNA-R (m) | ATTCACCCGACGGCATCTTT |
| CCND1-F (m) | AAAATGCCAGAGGCGGATGA |
| CCND1-R (m) | CAGGGCCTTGACCGGG |
| CCNA2-F (m) | TCTGGGATTAAAGGCGCCAC |
| CCNA2-R (m) | CCGACGGGAATCTTACAGGG |
| CCNE1-F (m) | GCTTCGGGTCTGAGTTCCAA |
| CCNE1-R (m) | GTCTTGCAAAAACACGGCCA |
| CCNB1-F (m) | ACAACGGTGAATGGACACCA |
| CCNB1-R (m) | GTACAGTTCAGCTGTGCCA |

**Table S2** Primers used Klf2 (rat) full-length cDNA amplification, and qRT-PCR

| Name | Sequence（5' to 3'） |
| --- | --- |
| Klf2-F (OE rat) | CCCAAGCTTGCCACCATGatggcgctcagcgagcctat |
| Klf2-R (OE rat) | CGCGGATCCctacatgtgtcgcttcatgtgcagagc |
| Klf2-F (rat) | CTTGCAGCTACACCAACTG |
| Klf2-R (rat) | TGATAAGGCTTCTCACCTGTG |
| c-Myc-F (rat) | CCCTACCCGCTCAACGACA |
| c-Myc-R (rat) | GCCTCTTTTCCACAGACACCA |
| c-Jun-F (rat) | ACAAACGTTGGTTGCGTGTT |
| c-Jun-R (rat) | GGCGAACCAAGGATTACGGA |
| PCNA-F (rat) | ACTTGGAATCCCAGAACAGG |
| PCNA-R (rat) | CACAGCATCTCCAATATGGC |
| CCNE1-F (rat) | TTCCTTATGGTGTCCTCGC |
| CCNE1-R (rat) | ACTGATAACCTGAGACCTTCTG |
| CCNB1-F (rat) | TCAGACAACTGGAGGAAGAG |
| CCNB1-R (rat) | GGATGGCTCTCATGTTTCC |

**Fig. S1** GO terms and KEGG pathways (p-value < 0.05) associated with the genes of cluster 2.

**(A)** GO analysis of functions of genes specifically enriched in cluster 2. **(B)** KEGG pathway enrichment analysis of inflammation-related signaling pathways enriched in cluster 2.

**Fig. S2** Analysis of chromatin accessibility of genes in different clusters at various early time points during liver regeneration. The chromatin accessibility profiles of select genes across the seven clusters. The chromatin accessibility changes within the range of 5 kb upstream to 5 kb downstream of genes were analyzed using ATAC-seq data.

**Fig. S3** Biological processes and related genes associated with the Foxo signaling pathway.

**Fig. S4** Motif analysis of transcription factor motif enrichment in the early stage of liver regeneration. **(A)** Motif analysis revealed a significant enrichment of the transcription factor Klf2's motif during the early phase of liver regeneration (0h_vs_2h). **(B)** Motif analysis revealed a significant enrichment of the transcription factor Klf2's motif during the early phase of liver regeneration (0h_vs_4h).

**Fig. S5** Association analysis of circadian rhythm-related gene expression with chromatin structure.

**(A)** The changes in Nr1d2 expression are consistent with its own chromatin interaction frequency (0h_vs_1h). **(B)** The changes in Per3 expression are consistent with its own chromatin interaction frequency (0h_vs_1h). **(C)** The changes in Rora expression are consistent with its own chromatin interaction frequency (0h_vs_1h). **(D)** The changes in Timeless expression are consistent with its own chromatin interaction frequency (0h_vs_1h). **(E)** The changes in Nfkbiz expression are consistent with its own chromatin interaction frequency (0h_vs_1h). **(F)** The changes in Dbp expression are consistent with its own chromatin interaction frequency (0h_vs_1h). **(G)** The changes in Cry2 expression are consistent with its own chromatin interaction frequency (0h_vs_1h).

**Fig. S6** Effects of Klf2 on AML12 cell viability.

**(A)** Quantitative real-time PCR of shKlf2 in AML12 cells. (**B)** Western blot of shKlf2 in AML12 cells. (**C)** MTT assay was performed to determine the viability of shKlf2 in AML12 cells. **(D)** Quantitative real-time PCR of Klf2 OE in AML12 cells. (**E)** Western blot of Klf2 OE in AML12 cells. (**F)** MTT assay was performed to determine the viability of Klf2-overexpressing AML12 cells. Data are representative of at least two independent experiments. * P <0.05, ** P <0.01 compared with control; Student's t -test.

**Fig. S7** Effects of Klf2 on BRL-3A cell proliferation and cell cycle progression.

**(A)** Quantitative real-time PCR of shKlf2 in BRL-3A cells. (**B)** Western blot of shKlf2 in BRL-3A cells. (**C)** MTT assay was performed to determine the viability of shKlf2 in BRL-3A cells. **(D**) CCK-8 assay was performed to determine the viability of shKlf2 in BRL-3A cells. **(E)** Quantitative real-time PCR of Klf2 OE in BRL-3A cells. (**F)** Western blot of Klf2 OE in BRL-3A cells. (**G)** MTT assay was performed to determine the viability of Klf2-overexpressing BRL-3A cells. (**H)** CCK-8 assay was performed to determine the viability of Klf2-overexpressing BRL-3A cells. **(I)** Quantitative real-time PCR detects the relative expression of cell proliferation and cell cycle related genes at the mRNA level after shKlf2. Data are representative of at least three independent experiments. **(J)** Quantitative real-time PCR detects the relative expression of cell proliferation and cell cycle related genes at the mRNA level after Klf2 OE. Data are representative of at least three independent experiments. * P <0.05, ** P <0.01, *** P <0.001 compared with control; Student's t-test.
